# Analysis of peptide PSY1 responding transcripts in the two *Arabidopsis* plant lines: wild type and psy1r receptor mutant

**DOI:** 10.1186/1471-2164-15-441

**Published:** 2014-06-06

**Authors:** Khalid Mahmood, Rubini Kannangara, Kirsten Jørgensen, Anja T Fuglsang

**Affiliations:** Department of Plant and Environmental Sciences, University of Copenhagen, Copenhagen, Denmark; PUMPKIN, Centre for membrane pumps in cells and disease, Copenhagen, Denmark

**Keywords:** Cellular functions, Gene enrichment analysis, Microarray, Signaling cascade, Small signaling peptides

## Abstract

**Background:**

Small-secreted peptides are emerging as important components in cell-cell communication during basic developmental stages of plant cell growth and development. Plant peptide containing sulfated tyrosine 1 (PSY1) has been reported to promote cell expansion and differentiation in the elongation zone of roots. PSY1 action is dependent on a receptor PSY1R that triggers a signaling cascade leading to cell elongation. However little is known about cellular functions and the components involved in PSY1-based signaling cascade.

**Results:**

Differentially expressed genes were identified in a wild type plant line and in a *psy1r* receptor mutant line of *Arabidopsis thaliana* after treatment with PSY1. Seventy-seven genes were found to be responsive to the PSY1 peptide in wild type plants while 154 genes were responsive in the receptor mutant plants. PSY1 activates the transcripts of genes involved in cell wall modification. Gene enrichment analysis revealed that PSY1-responsive genes are involved in responses to stimuli, metabolic processes and biosynthetic processes. The significant enrichment terms of PSY1-responsive genes were higher in *psy1r* mutant plants compared to in wild type plants. Two parallel responses to PSY1 were identified, differing in their dependency on the PSY1R receptor. Promoter analysis of the differentially expressed genes identified a light regulatory motif in some of these.

**Conclusion:**

PSY1-responsive genes are involved in cellular functions and stimuli responses suggesting a crosstalk between developmental cues and environmental stimuli. Possibly, two parallel responses to PSY1 exist. A motif involved in light regulation was identified in the promoter region of the differentially expressed genes. Reduced hypocotyl growth was observed in etiolated receptor mutant seedlings.

**Electronic supplementary material:**

The online version of this article (doi: 10.1186/1471-2164-15-441) contains supplementary material, which is available to authorized users.

## Background

In the past few years, our understanding of signals required for cell-to-cell communication during plant development has increased tremendously. Identification of components that mediate signaling serves as a landmark in understanding the mechanism of cell-to-cell communication in *planta*. Several components such as phytohormones, mobile transcription factors, mobile small RNAs and peptides serve this purpose [1, 2]. Phytohormones are lipophilic compounds which are active at very low concentrations and involved in plant growth ranging from embryogenesis to senescence [2]. Similarly, small-secreted peptides are now emerging as growth regulators and many of them are involved in basic functions of cell growth and development. More than 1000 genes are annotated as encoding putatively secreted peptides in the *Arabidopsis* genome but very few are known to be involved in specific cellular signaling [2, 3]. However, the precise role and mechanism proceeded by secreted peptides is yet to be established. Secreted peptides have now been recognized as a new class of intracellular signal molecules, which coordinate and specify cellular functions in plants. As a new class of intracellular signal molecules, the role of these secreted peptides could be explored further.

Plant cells transduce signals utilizing surface receptors binding to ligands present in the apoplast [4]. The S-locus receptor *ZmPK1* from Maize was the first receptor kinase identified in plants [5], and many receptors have been identified in plants ever since [6, 7]. Signaling molecules can elicit different signaling pathways, and a single receptor can also respond to more than one signal molecule [8]. Perception of signal molecules [9], and adjustability to different environmental conditions [10] are interesting characteristics of these signaling cascades. Plants demonstrate different growth patterns under different environmental conditions owing to asymmetric elongation or cell division [11–14]. They also exhibit flexibility in the size and numbers of produced organs to ensure diversity and specificity in perception of external stimuli [1, 2, 10–15].

Plant peptide containing sulfated tyrosine (PSY1) is a tyrosine-sulfated peptide isolated from *Arabidopsis* cell suspension medium [16]. It promotes cell expansion and differentiation in the elongation zone of roots at nanomolar concentration. This 18-amino acid glycopeptide is derived from a 75-amino acid precursor polypeptide containing an N-terminal signal peptide [16]. PSY1 is believed to bind the extracellular domain of Leucine rich Repeat Receptor Like Kinase (LRR-LK), which is named as a receptor of PSY1 (PSY1R). PSY1 and its receptor PSY1R are expressed throughout the whole plant with higher expression in shoot apical meristem and elongation zone of roots. PSY1 is known to be highly up-regulated after wounding [16]. Exogenous application of purified PSY1 peptide to suspension cell culture induces cellular proliferation, expansion and elongation while overexpression of *Arabidopsis* PSY1 causes longer roots with larger cotyledon as compared to wild type [16]. Recently, receptor PSY1R and peptide PSY1 were found to be involved in plant defense [17, 18]. The PSY1R might integrate growth promotion and defense signals leading to modulation of cellular plasticity, and may allow the cells to adjust towards environmental changes.

In order to understand the role of PSY1 and its receptor PSY1R, a full genome microarray study was performed. Identification of genes responding to PSY1 is a bottleneck in explaining the specific signaling phenomenon. This is the first comprehensive study to elucidate components of the PSY1-based signaling cascade using full genome microarray in response to exogenously applied PSY1. We found that several genes, involved in plethora of physiological functions, are differentially expressed after PSY1 exposure. Our study indicates that two PSY1 responses exist. The promoter analysis leads to identification of a light regulatory motif in differentially expressed genes of *psy1r* mutant plants.

## Results and discussion

### Genome wide analysis of two plant lines after PSY1 treatment and validation of microarray data

**Figure 1:**
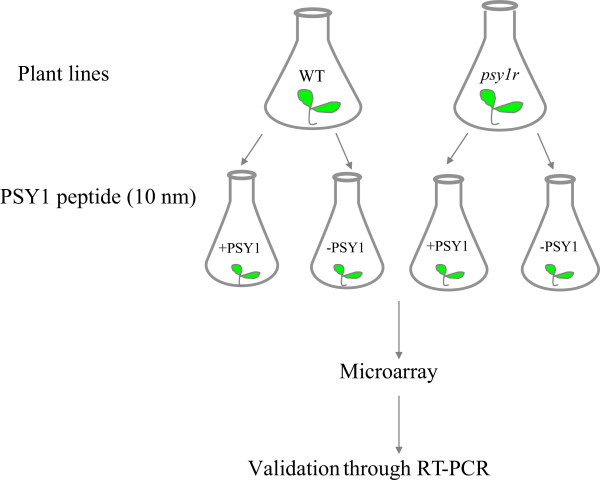
**Schematic presentation of experimental set**-**up.** Two plant lines were grown hydroponically for one week and following treated with 10 nM PSY1 for 4 hr before RNA extraction. Three independent biological samples were prepared.

In order to understand the PSY1-based signaling cascade, we decided to identify genes affected by PSY1. For this purpose, transcriptome analyses were performed on two plant lines after PSY1 treatment. The two plant lines (wild type and *psy1r*) were germinated and grown hydroponically for one week under sterile conditions. Then both lines were treated with PSY1 peptide (10 nM) for 4 hrs before mRNA isolation for microarray analysis (Figure 1). Three independent biological samples were prepared and an *Arabidopsis* Gene Expression Microarray (V4) of one color was used.

**Figure 2:**
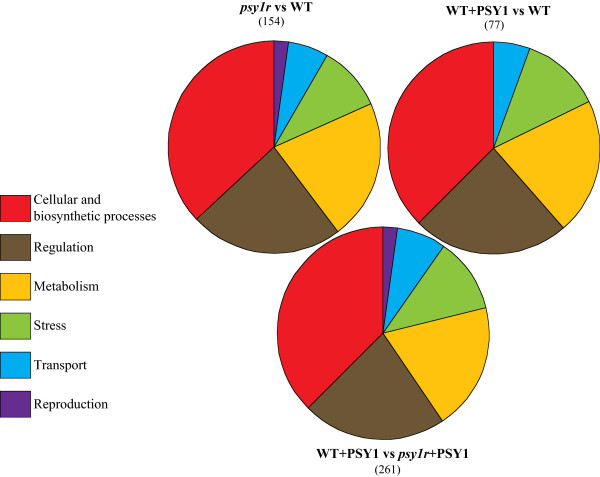
**Gene Ontology** **(** **GO** **)** **terms depicting the distribution of expressed genes by Amigo GO** **(** http://amigo.geneontology.org/cgi-bin/amigo/go.cgi **).** Venn diagram represents the percentage of each biological function obtained by genes involved in corresponding functions divided by all expressed genes. The category *psy1r* vs WT that represents the percentage of differentially expressed genes attributed to specific biological functions in *psy1r* mutant plants compared to wild type (*psy1r* vs WT). The category “WT + PSY1 vs WT” represents the percentage of differentially expressed genes after PSY1 treatment compared to untreated wild type. Other category “WT + PSY1 vs *psy1r* + PSY1” highlights the percentage of differentially expressed genes in PSY1-treated receptor mutant plants compared to PSY1-treated wild type plants.

In the transcriptome analysis, a number of genes were identified using the criteria; P < 0.05 and fold change >2 or < -2. Gene expression analysis after peptide treatment of wild type plants revealed significant differential expression of 77 genes. Among them 51 genes were up-regulated while 26 genes were down-regulated (Additional file 1: Table S1). Mutation of the receptor resulted in 154 genes with altered expression levels as compared to wild type (Figure 2). Among them, 102 genes were down-regulated while 52 genes were up-regulated in receptor knockout plants indicating the lack of a downstream response to the treatment (Additional file 2: Table S2). Interestingly, the number of differentially expressed genes (261) was higher when comparison was made between PSY1-treated wild type and PSY1-treated receptor knockout lines (*psy1r*) (Additional file 3: Table S3). Examination of Gene Ontology (GO) terms in both plant lines after PSY1 treatment demonstrated the specific contribution of different classes of biological functions in each category (Figure 2, Additional file 4: Table S4). Individual genes of each category were assigned to six different classes of relevant putative biological functions that were derived automatically using AmiGO. Interestingly all three comparisons showed the overall same distribution. Maximum numbers of differentially expressed genes were involved in cellular and biosynthetic processes while the major part of the genes were also involved in regulation and metabolism (Figure 2). The category “reproduction” was not found after PSY1 treatment in wild type while this category was present in receptor knockout plants (Figure 2). Additionally, the number of differentially expressed genes attributed as kinases or phosphatases were higher in receptor mutant plants as compared to PSY1 responsive genes (Additional file 1: Table S1 and Additional file 2: Table S2).

**Figure 3:**
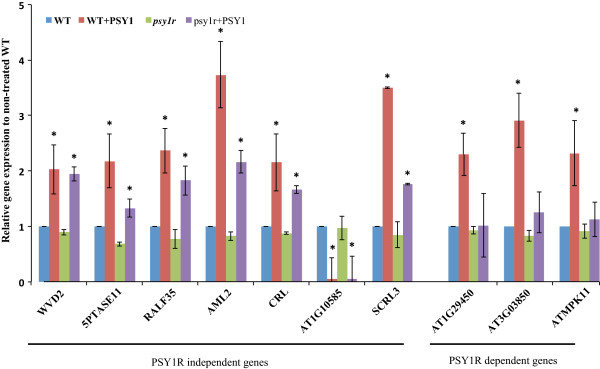
**Validation of microarray data through qRT-** **PCR of selected genes.** The transcript levels in plants were assessed following 4 h exposure of 10 nM of PSY1 to one-week-old plants. Relative gene expression levels were compared with wild type control (defined value of 1). The relative transcript levels were calculated from three independent biological replicates. * Indicates significance calculated among treatments using Tukey’s test (P < 0.05).

The expression levels found in the microarray study were confirmed by quantitative real time RT-PCR for selected genes (Figure 3). The genes were selected in view of their role in cell elongation with range of differential expression to validate microarray data. The selected genes included a novel protein WAVE-DAMPENED 2 (WVD2, AT5G28646) involved in cell expansion and root waving. Another gene that encodes a phosphatidylinositol polyphosphate 5-phosphatase (5PTase11, AT1G47510) also up-regulated after PSY1 treatment was selected, and so was RALF35, which is not characterized but belongs to another family of signaling peptide. Other tested proteins included AML2 (AT2G42890), which is involved in embryo development, CRL (AT5G51020), which is implicated in pattern of cell division and MPK11 (AT1G01560), which influences differentiation and plastid division. The specific primers used for each gene are listed in Additional file 5: Table S5. The qRT-PCR results generally agreed with the microarray data, however quantitative differences in expression levels were observed. In our experiments, the microarray was found to be more sensitive than the qRT-PCR data (Figure 3). Importantly, the qRT-PCR confirmed the presence of two groups of PSY1 responsive genes, one group dependent on the presence of PSY1R, the other group independent of PSY1R.

### Identification of enriched GO terms of differentially expressed genes in both plant lines

**Figure 4:**
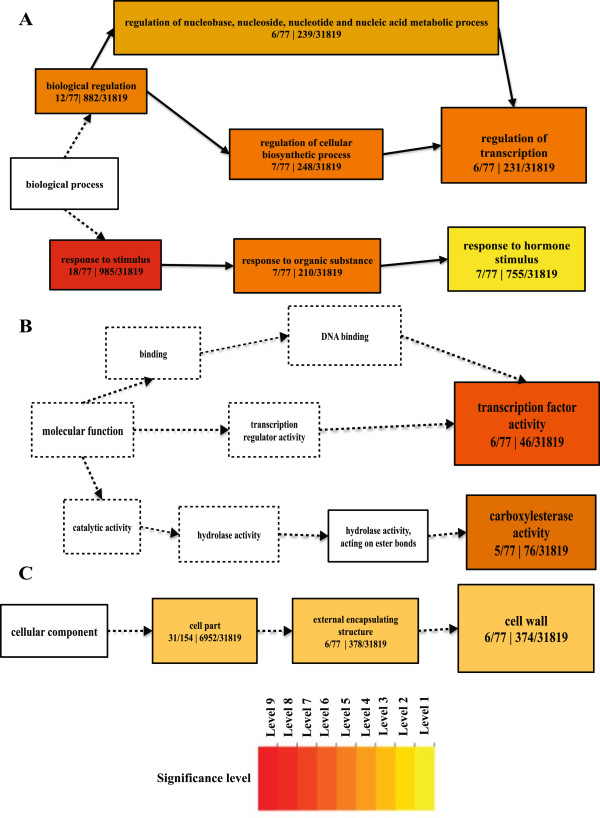
**GO-** **terms enrichments of differentially expressed genes in wild type after peptide PSY1 treatment.** The hierarchical graph of over-represented terms in Biological Processes **(A)**, Molecular Function **(B)** and Cellular Component **(C)** by Singular Enrichment Analysis (SEA) using AgriGO. Boxes in the graph represent GO term labeled by their GO ID, term definition and statistical information. The significant term (adjusted P < 0.05) is marked with color while non-significant terms are shown as white boxes. The boxes contain GO term labeled by their definition and numbers represent differentially expressed genes of a category divided by known total number of genes involved in specific GO term. The color-coding of a box represents the significance level. Solid, dashed and dotted lines represent two, one and zero enrichment terms at both ends connected by line, respectively. The rank direction of graph runs from left to right.

In order to identify enriched GO terms, an analysis of over-representing GO categories using AgriGO (Fisher’s exact test < 0.05) was conducted [19]. Over-representing GO terms were divided into Biological Processes (BP), Cellular Component (CC) and Molecular Function (MF). According to BP, 32 categories were significantly enriched in wild type after PSY1 treatment. To make it more simple and visual, the over-representing GO categories were drawn manually. These over-representing GO categories include response to hormone stimulus, regulation of biosynthetic processes and regulation of transcription (Figure 4A). When looking at cellular components (CC), four significant enriched categories were observed with majority of genes localized to cell wall (Figure 4B). Cell wall loosening requires lowering of the apoplastic pH as well as structural changes such as breakage of load-bearing bonds through enzymes [20]. According to molecular functions, two over-represented GO categories were found namely transcription factor activity and carboxylesterase activity (Figure 4C). Esterases remove methyl groups from polysaccharides and can thereby cause breakage of polysaccharides [21]. This is suggested to be one of the mechanisms required for cell elongation [22–24], and would support a mechanism of PSY1-induced cell growth.

**Figure 5:**
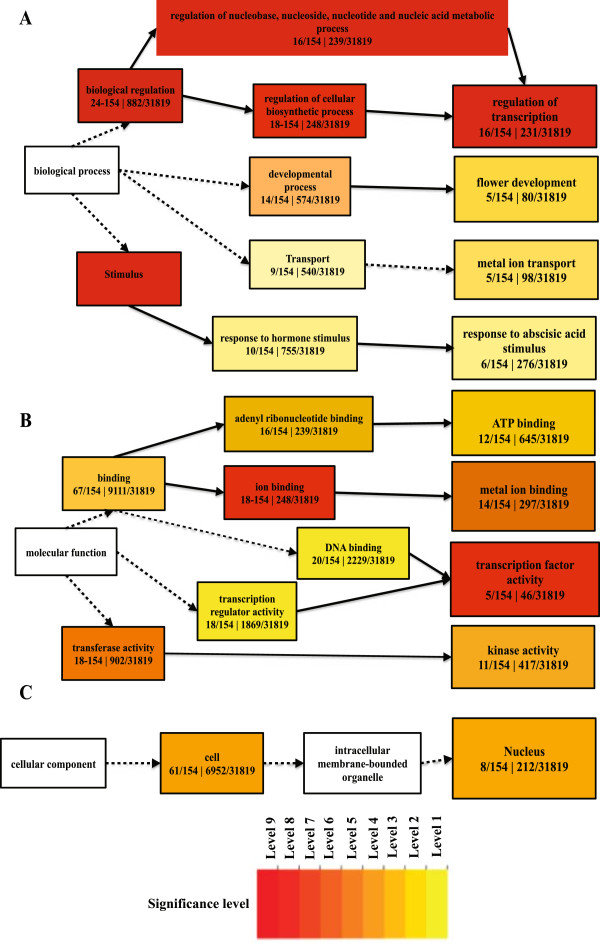
**GO-** **terms enrichments of differentially expressed genes in receptor knockout** **(** ***psy1r*** **)** **plants.** The hierarchical graph of over-represented terms in Biological Processes **(A)**, Molecular Function **(B)** and Cellular Component **(C)** by Singular Enrichment Analysis (SEA) using AgriGO. Boxes in the graph represent GO term labeled by their GO ID, term definition and statistical information. The significant term (adjusted P < 0.05) is marked with color while non-significant terms are shown as white boxes. The boxes contain GO term labeled by their definition and numbers represent differentially expressed genes of a category divided by known total number of genes involved in specific GO term. The color-coding of a box represents the significance level. Solid, dashed and dotted lines represent two, one and zero enrichment terms at both ends connected by line, respectively while color line represents negative correlation to the enrichment level of term. The rank direction of graph runs from left to right.

A functional enrichment study of differentially expressed genes in the *psy1r* mutant plants revealed that 52 GO terms were significantly enriched. The most prominent enriched functions in BP were response to stimuli, regulation of transcription, metal ion transport, flower development and response to abscisic acid stimulus (ABA) (Figure 5A). Response to stimuli and regulation of transcription are believed to happen through perception of PSY1 peptide by the receptor PSY1R as extracellular signals mediate specific cellular functions by triggering a signaling cascade that result in modulation of transcription factor activity [25]. Other significant enriched terms are metal ion transport genes (AT5G26690, AT2G28160, AT3G46900, AT3G48970 and AT5G52710) mainly involved in copper and iron transport. Both iron and copper transporters contribute to root elongation of *Arabidopsis thaliana*[26, 27]. The metal ion transport GO term’ is also found in the *psy1r* mutant plants. The GO term “response to abscisic acid stimulus” is enriched in *psy1r* mutant plants. Abscisic acid is involved in various processes including biotic and abiotic stress responses. A number of the differentially expressed genes (AT5G01540, AT3G28580, AT1G29395, AT1G73330 and AT1G48000) have previously been found to exhibit a negative regulation on abscisic acid response during growth [28]. Recently Mosher *et al*. (2013) found an involvement of PSY1R in plant defense [18]. This effect may be caused by changes in ABA levels, since ABA has profound roles in modulating diverse plant-pathogen interactions mediated by cross talk with the jasmonic acid and salicylic acid signal pathways [29]. Genes found in the *psy1r* mutant plants involved in GO term “flower development” (AT4G16280, AT5G67060, AT1G24260, AT4G01500 and AT3G50330) are mainly involved in promotion of transition of vegetative meristem to reproductive development, carpel formation and ovule development [30–32]. According to cellular component (CC), over-representing GO term found in the *psy1r* mutant plant is nucleus (Figure 5B). This is not surprising due to the increase in transcription factor activity occurring in the nucleus. According to molecular functions (MF), 22 categories were significantly enriched. The most significant enriched functions were ion binding followed by transcription factor activity, kinase activity and ATP binding (Figure 5C). These molecular function GO terms make sense in *psy1r* mutant plants due to PSY1R role in these functions and being a receptor of peptide PSY1.

In the PSY1-treated *psy1r* mutant plants, 62 GO terms were found according to biological component (BP) and these numbers were higher in the PSY1-treated *psy1r* mutant plants than in untreated *psy1r* mutant plants. GO terms lacking in the untreated *psy1r* mutant is “response to auxin stimulus” and “cellular amino acid metabolic processes”. No differences were observed according to cellular component. When comparing molecular function GO terms of PSY1-treated and untreated *psy1r* plants, most prominent difference is the carboxylesterase activity observed in peptide-treated plants. This enzyme activity is completely absent in the untreated mutant plant line. These differences encouraged a more comprehensive analysis of differentially expressed genes in the two plant lines.

### Receptor dependent and independent response to peptide PSY1

**Figure 6:**
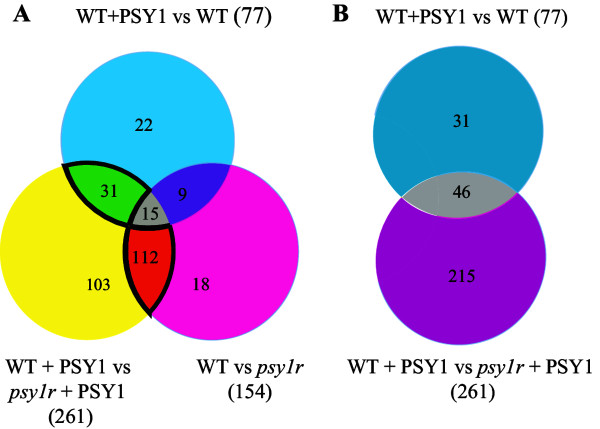
**Comparison among differentially expressed genes in both plant lines with and without peptide treatment. A)** Venn diagram comparing differentially expressed genes in all three treatments. Genes marked “Bright green” are common between both plant lines after PSY1 treatment, genes marked “gray” are differentially expressed in all three treatments. Genes marked with “violet color” are common between peptide-treated wild type and untreated *psy1r* mutant plants. Genes marked with “yellow”, “blue color” and “pink color” are solely expressed in one treatment but absent in the other treatments. The 112 genes marked with “orange” are common between treated and untreated *psy1r* mutant plants. **B)** Venn diagram of PSY1-responsive genes. Grey: genes differentially expressed in both plant lines after PSY1 treatment, blue and pink represents genes induced in wild type and in *psy1r* mutant plants, respectively, after PSY1 exposure.

In order to compare the affected genes Venn diagrams were drawn. The diagrams provide a comparison of differentially expressed genes in all three treatments (Figure 6A). The diagrams illustrate that some differentially expressed genes are common among treatments and some solely expressed in one treatment but absent in other treatments. Additionally a simplified comparison is made between peptide-treated wild type and peptide-treated *psy1r* mutant plants (Figure 6B). It revealed that PSY1 does trigger a response even in the absence of PSY1R, suggesting the existence of two parallel responses, named as a) PSY1R-independent response and b) PSY1R-dependent response. The genes that responded to PSY1 in both plant lines belongs to the PSY1R-independent response, while the PSY1R dependent response consists of genes that are differentially expressed as response to PSY1 only in the presence of the receptor PSY1R. The PSY1R-receptor independent response includes 46 genes expressed in both plant lines after PSY1 treatment (Figure 6B). There could be two possible reasons for transcripts expression in *psy1r*-independent response. The first one might be due to absence of natural receptor PSY1R, PSY1 binds to a low affinity receptor that capable of activating another signaling cascade results in transcripts expression. Alternatively, second one may explain that the addition of exogenous PSY1 peptide in the presence of endogenous PSY1 may cause homeostatic disturbance and a parallel signaling cascade is initiated. Nevertheless, a clear response is observed after exogenous peptide treatment.

Interestingly, when analyzing the over-represented GO categories it is found that the PSY1R-independent pathway represents the cell wall modifications (hydrolase activity), while the PSY1R-dependent pathway represents the response to stimuli, regulation of transcription, metal ion transport, flower development and response to abscisic acid stimulus.

### Identification of light-responsive *cis* elements in promoters of differentially expressed genes

**Figure 7:**
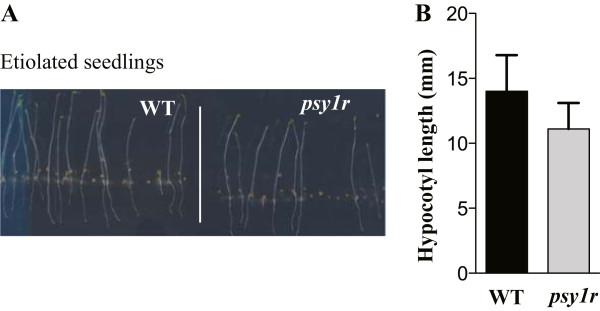
**Wild type** (**WT**) **and** ***psy1r*** **mutant seedlings grown for 5 days in the dark. A)** Seeds of wild type and *psy1r* mutant plants were grown ½ MS + 1% sucrose agar plates. Results are average ((±SE) of minimum of 92 hypocotyls (Wt n = 108, *psy1r* n = 92 analyzed per genotype (P < 0.001 ANOVA, Tukey test). **B)** Hypocotyl length (mm) of representative WT and *psy1r* mutant plant grown in dark.

A search for common elements (*cis* elements) in the PSY1 affected genes was carried out using the Promomer tools in the Botany Array Resource [33]. The Promomer tools use alignment of sequences of genes and enumerative method to find motifs. We searched for over-represented 6-bp motifs in the 1-kb upstream promoter region (Table 1). The data indicated that all putative motifs are TA rich. It is known that TA rich sequences in the core promoter region act in promoting or repressing genes at the transcriptional level [34]. The highly significant motifs include TATATA and TGTATA (Table 1), which are a part of the light regulatory motif (TGTATATAT). The TGTATATAT motif was previously shown to be involved in the network of light regulated genes [35]. This motif is found in the promoter region of 14 genes of *psy1r* mutant plants and in only two PSY1-responsive gene promoters in wild type plants, accounting for 9% and 2% of all differentially expressed genes, respectively (Table 1). The fourteen genes of *psy1r* mutant plants containing the light regulatory motif in the promoter region are: Isochorismate synthase 2 (ICS2, AT1G18870), Knox *Arabidopsis thaliana* meinox (KNATM, AT1G14760), RXW8 (AT1G58520), AT1G23205, Ovate family protein 16 (OFP16, AT2G32100), AT3G62990, Cytochrome P450 (CYP82C2, AT4G31970), NGA4 (AT4G01500), Peroxidase 52 (PRX52, AT5G05340), Sweet 12 (AT5G23660), AT5G46080, NDR1-like 3 (AT5G06320), Longifolia 1 (AT5G15580) and AT5G21910. The Longifolia 1 is known to regulate longitudinal cell elongation in *Arabidopsis thaliana*[36]. The finding of TGTATATAT in differentially expressed genes of *psy1r* mutant plants may suggest an involvement of PSY1R in light response. A separate experiment was conducted in order to test if *psy1r* knockout plants react differently to the lack of light. Elongation of hypocotyls of etiolated *psy1r* plants was analyzed after 5 days of growth. Comparison of hypocotyls of dark- grown plants revealed shorter hypocotyls in *psy1r* mutant plants compared to wild type (Figure 7). These results also support the view that PSY1R contributes to hypocotyl cell elongation in response to light.

**Table 1:** **Putative** ***cis***-**regulatory motives statistically over**-**represented in the promoters of differentially expressed genes**

Motif	PSY1 responsive genes in wild type (77 genes)				Differentially expressed genes in ***psy1r*** mutant plants (154 genes)				Differentially expressed genes in PSY1 treated wild type plants compared to PSY treated ***psy1r*** mutant plants (261 genes)			
	Hits out of 77 genes	%	Z-score	p-value	Hits out of 154 genes	%	Z-score	p-value	Hits out of 261 genes	%	Z-score	p-value
**TATATA**	58	75	2.1	0.05	141	92	5.7	0.001	221	85	3.6	0.001
**AAAATA**	75	97	2.0	0.05	149	97	3.6	0.001	256	98	3.5	0.001
**AAAATT**	74	96	2.0	0.05	148	96	1.4	ns	254	97	2.6	0.005
**AATAAA**	76	99	1.7	0.05	148	96	3.1	0.001	253	97	3.8	0.001
**TGTATA**	53	68	1.7	0.05	114	74	3.0	0.001	199	76	3.2	0.001
**TGTATATAT**	2	2	ns	ns	14	9	ns	ns	21	8	ns	ns

These sequences have been obtained using the programs Promomer (http://bar.utoronto.ca/ntools/cgi-bin/BAR_Promomer.cgi) and Motif Analysis (TAIR). Z-scores and p-values are those calculated by the corresponding program (Promomer or Motif Analysis).

## Conclusions

Small signaling peptides perceived by receptors are situated in specific cells to control growth and development by eliciting a vast array of physiological responses. PSY1 is a secreted peptide and its action is dependent on a receptor PSY1R that triggers a signaling cascade leading to cell elongation. However, the targets of this signaling pathway are yet to be studied in detail. Our work revealed that addition of exogenous PSY1 leads to transcription of cell wall modifying enzymes, enzymes that might contribute to the loosening of the cell wall during elongation. Two parallel responses to PSY1 were found to exist (PSY1R-dependent and PSY1R-independent). This could suggest that other receptors for PSY1 peptides exist within the plant. PSY1-responsive genes encode several genes localized in cell wall that regulate carboxylestrase activity, while differentially expressed genes in *psy1r* mutant plants largely were localized to the nucleus with molecular function of ion binding and transcription factor activity. A major part of PSY1-responsive genes were involved in cell growth, cell differentiation and catabolic processes. Genome wide gene expression profiling based on GO revealed that most differentially expressed genes were involved in cellular functions and response to stimuli. This suggests a crosstalk between developmental cues and environmental stimuli. A promoter analysis revealed a specific *cis*-element present in 9% of the differentially expressed genes of *psy1r* mutant plants. This element has previously been found in genes regulated by light. Elongation growth of hypocotyls is closely linked to light and one can speculate that PSY1R is involved in the regulation of light response.

## Methods

### Plant material and growth conditions

Experiments with *Arabidopsis thaliana* were performed on ecotype Columbia-0. Twenty-five milligram (mg) of seeds of two plant lines (wild type and *psy1r* knockout) were surface sterilized in a micro-centrifuge tube by treating them with ethanol and subsequently Klorin containing 0.2% tween for 10 min. Seeds were then washed again with ethanol for 10 min and rinsed with sterile water twice before subjecting them to imbibition and stratification at 4°C for three days. After stratification, seeds of the two plant lines were grown hydroponically.

Nutrient solution of hydroponic culture containing half strength of Murashige and Skoog medium (MS) with 1% (w/v) sucrose was prepared (pH 5.7, KOH) and sterilized by autoclaving. The stratified seeds of the two plant lines were grown in 500 mL-conical flasks containing 250 mL nutrient solution. Flasks were placed in a growth chamber on a shaker at 180 rpm under continuous light and sterilized conditions. The purified, natural PSY1 was obtained from Yoshikatsu Matsubyashi Lab, National Institute for basic biology, Japan and PSY1 applied to seedlings as described by Amano et al [16]. After one week, plants were treated with the PSY1 peptide at 10 nM concentration for 4 hrs and then transferred into liquid nitrogen and stored at -80°C until RNA extraction.

### RNA extraction and microarray

Total RNA was extracted from three biological replicates of 25 mg seedlings grown in hydroponic culture in sterile conditions using the RNeasy Plant mini kit (Qiagen). RNA integrity was assessed using an Agilent 2100 Bioanalyser with RNA 6000 Nano Assay (Agilent Technologies) and was processed for use on Arabidopsis (V4) Gene Expression Microarray (Agilent Technologies). Arabidopsis (V4) Gene Expression Microarray was used for RNA analysis according to manufacturer’s detail (Design ID: 21169, G2519F; Agilent Technologies, Palo Alto, CA, USA). Briefly, 200 ng of total RNA containing RNA spiked in Mix was reverse transcribed in to cDNA that was then *in vitro* transcribed into cRNA, labeled with cyanine 3-CTP using Agilent Low RNA Input Linear Amp Kit (Agilent Technologies). The Agilent RNA spike-in control targets are a set of 10 *in vitro*-synthesized poly-adenylated transcripts derived from the adenovirus E1A gene used to monitor the labeling reactions and the microarray performance. For labeled cRNA purification, 84 μl sterile H_2_O, 350 μl RLT buffer from Qiagen RNAeasy Mini Kit (Qiagen Technologies), and 250-μl EtOH were added. The purification steps followed the protocol described by the manufacturer.

After obtaining the required cRNA yield and incorporation rate of fluorescent dye cyanine 3-CTP, a hybridization step was carried out simultaneously for all three biological repeats. Hybridizations were carried out in Agilent’s SuperHyb Hybridization Chambers (Agilent Technologies) containing 5 μg of cyanine 3-labeled linearly amplified cRNA. The hybridization reaction was performed at 65°C for 17 hours using the Agilent DNA microarray hybridization oven (Agilent Technologies), following procedures described in the Agilent One-Color Microarray-Based Gene Expression Analysis protocol. The hybridized microarrays were disassembled in Agilent Gene Expression Wash Buffer 1 (Agilent Technologies) and then washed with the same buffer for 1 min at room temperature, followed by washing with Gene Expression Wash Buffer 2 for one min at 37°C. The microarrays were then scanned immediately using the Agilent DNA Microarray Scanner (Agilent Technologies). The images generated were analyzed with Agilent Feature Extraction Software. The raw data of hybridization was imported into the microarray analysis software GeneSpring 11.5 (Agilent Technologies). Normalization and background intensity determination for each feature performed using the Robust Multiarray Average summarization algorithm, as described by Irizarry et al. [37]. Genes were considered differentially regulated if their normalized expression value was significantly different from the control (*P* < 0.05). One-way ANOVA with Benjamini Hochberg multiple testing corrections (false discovery rate of 0.05) was used to identify genes differentially regulated between treatment groups. Genes exhibiting more than a 2-fold enhanced or reduced transcription level in three independent experiments were considered to show significant alterations in expression.

### Real time PCR

Aliquots of RNA samples used for the microarray analysis were also analyzed by real-time RT-PCR. Reverse transcription (RT) was performed with 2 μg of total RNA to obtain cDNA with SuperScript II and Oligo (dT)12-18 (Invitrogen) as the primer in a 20 μl reaction volume. Each cDNA sample was diluted 1:4 in sterile ddH2O, and 1 μl of this dilution was used as template for qPCR. Primers for the PCR reactions were designed by Beacon Designer™ to have a Tm of ~ 60°C and an optimal annealing temperature of 53–55°C with the length of the amplicons between 120 and 300 bp. Real-time PCR was performed with DyNAmo™ Flash SYBR® Green qPCR Kit (Qiagen) in 20 μL reactions according to manufacturer’s instruction. Each PCR reaction contains 5 μl of diluted cDNA (100 ng), 5 μl (0.5 μM) of both primers and 10 μl of DyNAmo™ Flash SYBR® Green master mix. The initial denaturing time was 7 min at 95°C, followed by 45 cycles consisting of 95°C for 10 s, 57°C for 15 s, 68°C for 30 s and 75°C for 1 s with a single fluorescence measurement. Then it was held at 60°C for 60s. A melting curve analysis of the generated products (65°C–95°C with a heating rate of 1°C s^-1^ and a continuous fluorescence measurement) was performed after the PCR cycles.

*ACT2* (AT3G18780) was selected as a valid housekeeping gene since the expression of *ACT2* did not change significantly in plant lines treated with the PSY1 peptide compared to untreated plant lines (Additional file 6: Figure S1). In addition to this, no significant changes in expression of *ACT2* could be observed in the microarray data further demonstrating that the *ACT2* expression is unaffected by the PSY1 peptide treatment. For relative quantification, amplification efficiencies (E) for primer set targeting each gene were determined in the following way: an aliquot of cDNA transcribed from 5 μg of total RNA was diluted with sterile ddH_2_O to 10^-1^, 10^-2^ and 10^-3^. Standard curves for each gene were performed using the undiluted and diluted cDNA to cover the range of all template concentrations. The specific primers for each gene were used. Gene-specific PCR efficiency was used to calculate the expression of target genes relative to the expression of *ACT2* reference gene. The ∆CT value was calculated as follows: ∆CT (target genes) = CT (target gene)- CT (Reference gene), where CT is the cycle number at which PCR product exceeded a set threshold. Relative transcript level (RTL) was calculated through = 1× 2^-∆CT^.

### Gene ontology

Gene ontologies were analyzed for term enrichment using the AgriGO Single Enrichment Analysis tool with TAIR10 GO annotation (http://bioinfo.cau.edu.cn/agriGO/). GO enrichment was performed in AgriGO (FDR correction and Fisher’s exact test < 0.05) using the whole *Arabidopsis* genome as the background/reference.

### Hypocotyl length measurements in dark grown plants

Seedlings of *psy1r* and wild type plants were grown on MS medium (0.8% (w/v) agar and 1% (w/v) sucrose) at 22°C for 5 days. Seedlings were transferred to transparencies, scanned and measured using the application ImageJ for hypocotyl length measurements.

### Availability of supporting data

The data sets supporting the results of this article are included within the article (and its additional files along with list of genes). Raw microarray data were deposited to GEO public database and available under the accession number “GSE55684”. (http://www.ncbi.nlm.nih.gov/geo/query/acc.cgi?acc=GSE55684).

## Electronic supplementary material

Additional file 1: Table S1: List of genes that were differentially expressed in wild type plants after PSY1 treatment. Genes were identified using the criteria; P < 0.05 and fold change >2 or < -2 through One-way ANOVA (with Benjamini Hochberg multiple testing corrections and FDR < 0.05) between control and peptide PSY1 treated wild type plants. The up-regulated and down regulated genes were sorted from highest to lowest fold expression values. (DOCX 110 KB)

Additional file 2: Table S2: List of all genes differentially expressed in *psy1r* mutant plant compared to wild type plants. Genes were identified using the criteria; P < 0.05 and fold change >2 or < -2 through One-way ANOVA (with Benjamini Hochberg multiple testing corrections and FDR < 0.05) between *psy1r* mutant plants and wild type plants. The up-regulated and down regulated genes were sorted from highest to lowest fold expression values. (DOCX 112 KB)

Additional file 3: Table S3: List of genes differentially expressed in PSY1-treated *psy1r* mutant plants compared to peptide treated wild type plants. Genes were identified using the criteria; P < 0.05 and fold change >2 or < -2 through One-way ANOVA (with Benjamini Hochberg multiple testing corrections and FDR < 0.05) between PSY1 treated *psy1r* mutant plants and PSY1 treated-wild type plants. The up-regulated and down regulated genes were sorted from highest to lowest fold expression values. (DOCX 120 KB)

Additional file 4: Table S4: Functional grouping of genes showing differential transcript expression in different treatments. Gene ontology (GO) enrichment was performed in AgriGO (FDR correction and Fisher’s exact test < 0.05) using the whole *Arabidopsis* genome as the background/reference. (DOCX 25 KB)

Additional file 5: Table S5: Primers used for RT-PCR validation of selected genes identified by microarray after PSY1 treatment in wild type plants. (DOCX 19 KB)

Additional file 6: Figure S1: The *ACT2* (At3g18780) gene expression. The expression of *ACT2* was not significantly altered in both plant lines treated with the PSY1 peptide compared to the untreated plant lines. The significance level was tested among three independent biological replicates (n = 3). The scale bars represent standard error (S.E) among three biological samples. (PDF 383 KB)

## Abbreviations

PSY1: Plant peptide containing sulfated tyrosine 1
PSY1R: Receptor of plant peptide containing sulfated tyrosine 1
RNA: Ribonucleic acid
LRR-LK: Leucine rich repeat receptor like kinase
GO: Gene Ontology
RT-PCR: Real time polymerase chain reaction
WVD2: Wave dampened2
BP: Biological processes
CC: Cellular component
MF: Molecular function
ABA: Abscisic acid
MS: Murashige and Skoog medium
RT: Reverse transcription
RTL: Relative transcript level
SEA: Singular enrichment analysis
ANOVA: Analysis of variance.
