# Supplementary material for: Analysis of peptide PSY1 responding transcripts in the two Arabidopsis plant lines: wild type and psy1r receptor mutant
Source: BMC Genomics. 2014 Jun 6;15(1):441. doi: 10.1186/1471-2164-15-441 (PMC4070568; doi:10.1186/1471-2164-15-441)
Supplement: Supplementary file 1 — Additional file 1: Table S1: List of genes that were differentially expressed in wild type plants after PSY1 treatment. Genes were identified using the criteria; P < 0.05 and fold change >2 or < -2 through One-way ANOVA (with Benjamini Hochberg multiple testing corrections and FDR < 0.05) between control and peptide PSY1 treated wild type plants. The up-regulated and down regulated genes were sorted from highest to lowest fold expression values. (DOCX 110 KB) [file 12864_2013_6150_MOESM1_ESM.docx]

**Supplementary table 1: List of genes that were differentially expressed in wild type plants after PSY1 treatment**

| **Gene Name** | **Gene Locus** | **Fold change** | **Regulation** |
| --- | --- | --- | --- |
| SCRL3 (SCR-Like 3) | [AT1G08695](http://www.arabidopsis.org/servlets/TairObject?type=locus&name=AT1G08695) | 17.63 | up |
| F-box family protein-related | [AT1G64295](http://www.arabidopsis.org/servlets/TairObject?type=locus&name=AT1G64295) | 7.12 | up |
| WVD2 | [AT5G28646](http://www.arabidopsis.org/servlets/TairObject?type=locus&name=AT5G28646) | 6.62 | up |
| BAM6 (BETA-AMYLASE 6); beta-amylase/ catalytic/ cation binding | [AT2G32290](http://www.arabidopsis.org/servlets/TairObject?type=locus&name=AT2G32290) | 5.96 | up |
| SCRL17 (SCR-Like 17) | [AT2G25685](http://www.arabidopsis.org/servlets/TairObject?type=locus&name=AT2G25685) | 4.57 | up |
| 5PTASE11 (INOSITOL POLYPHOSPHATE 5-PHOSPHATASE 11) | [AT1G47510](http://www.arabidopsis.org/servlets/TairObject?type=locus&name=AT1G47510) | 3.99 | up |
| hypothetical protein | [AT2G32785](http://www.arabidopsis.org/servlets/TairObject?type=locus&name=AT2G32785) | 3.98 | up |
| protease inhibitor/seed storage/lipid transfer protein (LTP) family protein | [AT5G55450](http://www.arabidopsis.org/servlets/TairObject?type=locus&name=AT5G55450) | 3.62 | up |
| nodulin MtN21 family protein | [AT1G68170](http://www.arabidopsis.org/servlets/TairObject?type=locus&name=AT1G68170) | 3.09 | up |
| heavy-metal-associated domain-containing protein | [AT5G52710](http://www.arabidopsis.org/servlets/TairObject?type=locus&name=AT5G52710) | 2.70 | up |
| PDF1.2b (plant defensin 1.2b) | [AT2G26020](http://www.arabidopsis.org/servlets/TairObject?type=locus&name=AT2G26020) | 2.58 | up |
| AML2 (ARABIDOPSIS-MEI2-LIKE 2) | [AT2G42890](http://www.arabidopsis.org/servlets/TairObject?type=locus&name=AT2G42890) | 2.58 | up |
| GDSL-motif lipase/hydrolase family protein | [AT5G08460](http://www.arabidopsis.org/servlets/TairObject?type=locus&name=AT5G08460) | 2.56 | up |
| ATGPX7 (glutathione peroxidase 7); glutathione peroxidase | [AT4G31870](http://www.arabidopsis.org/servlets/TairObject?type=locus&name=AT4G31870) | 2.54 | up |
| hypothetical protein | [AT5G44650](http://www.arabidopsis.org/servlets/TairObject?type=locus&name=AT5G44650) | 2.50 | up |
| lipase class 3 family protein | [AT4G16070](http://www.arabidopsis.org/servlets/TairObject?type=locus&name=AT4G16070) | 2.49 | up |
| GRAM domain-containing protein / ABA-responsive protein-related | [AT4G01600](http://www.arabidopsis.org/servlets/TairObject?type=locus&name=AT4G01600) | 2.46 | up |
| phosphoinositide binding | [AT3G63300](http://www.arabidopsis.org/servlets/TairObject?type=locus&name=AT3G63300) | 2.45 | up |
| isochorismatase hydrolase family protein | [AT3G16190](http://www.arabidopsis.org/servlets/TairObject?type=locus&name=AT3G16190) | 2.41 | up |
| auxin-responsive protein, putative" | [AT1G29450](http://www.arabidopsis.org/servlets/TairObject?type=locus&name=AT1G29450) | 2.39 | up |
| SULTR3;1 (SULFATE TRANSPORTER 3;1) | [AT3G51895](http://www.arabidopsis.org/servlets/TairObject?type=locus&name=AT3G51895) | 2.33 | up |
| hypothetical protein | [AT2G27290](http://www.arabidopsis.org/servlets/TairObject?type=locus&name=AT2G27290) | 2.30 | up |
| hypothetical protein | [AT1G16850](http://www.arabidopsis.org/servlets/TairObject?type=locus&name=AT1G16850) | 2.30 | up |
| protease inhibitor/seed storage/lipid transfer protein (LTP) family protein | [AT5G55460](http://www.arabidopsis.org/servlets/TairObject?type=locus&name=AT5G55460) | 2.27 | up |
| ADF4 (ACTIN DEPOLYMERIZING FACTOR 4); actin binding | [AT5G59890](http://www.arabidopsis.org/servlets/TairObject?type=locus&name=AT5G59890) | 2.26 | up |
| hypothetical protein | [AT3G27050](http://www.arabidopsis.org/servlets/TairObject?type=locus&name=AT3G27050) | 2.26 | up |
| GCN5-related N-acetyltransferase (GNAT) family protein | [AT4G28030](http://www.arabidopsis.org/servlets/TairObject?type=locus&name=AT4G28030) | 2.24 | up |
| PDF1.3 (plant defensin 1.3) | [AT2G26010](http://www.arabidopsis.org/servlets/TairObject?type=locus&name=AT2G26010) | 2.23 | up |
| PDF1.2c (plant defensin 1.2c) | [AT5G44430](http://www.arabidopsis.org/servlets/TairObject?type=locus&name=AT5G44430) | 2.21 | up |
| UBX domain-containing protein | [AT3G21660](http://www.arabidopsis.org/servlets/TairObject?type=locus&name=AT3G21660) | 2.21 | up |
| strictosidine synthase family protein | [AT3G51450](http://www.arabidopsis.org/servlets/TairObject?type=locus&name=AT3G51450) | 2.19 | up |
| misc_RNA | [AT5G13887](http://www.arabidopsis.org/servlets/TairObject?type=locus&name=AT5G13887) | 2.16 | up |
| hypothetical protein | [AT3G07310](http://www.arabidopsis.org/servlets/TairObject?type=locus&name=AT3G07310) | 2.14 | up |
| LCV2 (LIKE COV 2) | [AT1G43130](http://www.arabidopsis.org/servlets/TairObject?type=locus&name=AT1G43130) | 2.14 | up |
| hypothetical protein | [AT3G62580](http://www.arabidopsis.org/servlets/TairObject?type=locus&name=AT3G62580) | 2.14 | up |
| DNA polymerase V family | [AT5G64420](http://www.arabidopsis.org/servlets/TairObject?type=locus&name=AT5G64420) | 2.13 | up |
| auxin-responsive protein, putative" | [AT3G03850](http://www.arabidopsis.org/servlets/TairObject?type=locus&name=AT3G03850) | 2.11 | up |
| haloacid dehalogenase-like hydrolase family protein | [AT1G14310](http://www.arabidopsis.org/servlets/TairObject?type=locus&name=AT1G14310) | 2.11 | up |
| hypothetical protein | [AT4G17250](http://www.arabidopsis.org/servlets/TairObject?type=locus&name=AT4G17250) | 2.10 | up |
| PPT2 (PHOSPHOENOLPYRUVATE (PEP)/PHOSPHATE TRANSLOCATOR 2 | [AT3G01550](http://www.arabidopsis.org/servlets/TairObject?type=locus&name=AT3G01550) | 2.08 | up |
| aminoacyl-tRNA hydrolase/ protein tyrosine phosphatase | [AT5G10700](http://www.arabidopsis.org/servlets/TairObject?type=locus&name=AT5G10700) | 2.05 | up |
| misc_RNA | [AT1G66173](http://www.arabidopsis.org/servlets/TairObject?type=locus&name=AT1G66173) | 2.04 | up |
| hypothetical protein | [AT5G44005](http://www.arabidopsis.org/servlets/TairObject?type=locus&name=AT5G44005) | 2.04 | up |
| hypothetical protein | [AT5G44005](http://www.arabidopsis.org/servlets/TairObject?type=locus&name=AT5G44005) | 2.04 | up |
| CRL (CRUMPLED LEAF) | [AT5G51020](http://www.arabidopsis.org/servlets/TairObject?type=locus&name=AT5G51020) | 2.03 | up |
| misc_RNA | [AT5G44562](http://www.arabidopsis.org/servlets/TairObject?type=locus&name=AT5G44562) | 2.03 | up |
| hypothetical protein | [AT3G21310](http://www.arabidopsis.org/servlets/TairObject?type=locus&name=AT3G21310) | 2.03 | up |
| TAF2 (TBP-ASSOCIATED FACTOR 2); metallopeptidase/ zinc ion binding | [AT1G73960](http://www.arabidopsis.org/servlets/TairObject?type=locus&name=AT1G73960) | 2.02 | up |
| hypothetical protein | [AT2G41120](http://www.arabidopsis.org/servlets/TairObject?type=locus&name=AT2G41120) | 2.01 | up |
| hypothetical protein | [AT3G43572](http://www.arabidopsis.org/servlets/TairObject?type=locus&name=AT3G43572) | 2.01 | up |
| ATMPK11; MAP kinase/ kinase | [AT1G01560](http://www.arabidopsis.org/servlets/TairObject?type=locus&name=AT1G01560) | 2.01 | up |
| basic helix-loop-helix (bHLH) family protein | AT4G20970 | 7.90 | down |
| transcription factor | AT1G10585 | 6.39 | down |
| AtGolS6 (Arabidopsis thaliana galactinol synthase 6) | AT4G26250 | 4.04 | down |
| MYB112 (myb domain protein 112); DNA binding / transcription factor | AT1G48000 | 3.71 | down |
| peroxidase, putative | AT5G05340 | 3.48 | down |
| DIN2 (DARK INDUCIBLE 2) | AT3G60140 | 3.40 | down |
| hypothetical protein | AT3G62990 | 3.37 | down |
| triacylglycerol lipase | AT5G24200 | 3.26 | down |
| MPC (MATERNALLY EXPRESSED PAB C-TERMINAL); poly(A) binding | AT3G19350 | 2.79 | down |
| MYB7 (MYB DOMAIN PROTEIN 7); DNA binding / transcription factor | AT2G16720 | 2.75 | down |
| oxidoreductase, 2OG-Fe(II) oxygenase family protein" | AT3G55970 | 2.68 | down |
| pectinesterase family protein | AT5G20860 | 2.64 | down |
| hypothetical protein | AT3G62990 | 2.61 | down |
| ATTI1; serine-type endopeptidase inhibitor | AT2G43510 | 2.50 | down |
| DREB2A; DNA binding / transcription activator/ transcription factor | AT5G05410 | 2.48 | down |
| ethylene-responsive factor, putative" | AT4G18450 | 2.39 | down |
| SAP domain-containing protein | AT5G66840 | 2.35 | down |
| NADP-dependent oxidoreductase, putative" | AT5G16960 | 2.27 | down |
| meprin and TRAF homology domain-containing protein | AT2G05400 | 2.18 | down |
| hypothetical protein | AT5G01740 | 2.17 | down |
| CYP72A13; electron carrier | AT3G14660 | 2.11 | down |
| hypothetical protein | AT4G07740 | 2.05 | down |
| ICL (ISOCITRATE LYASE); catalytic/ isocitrate lyase | AT3G21720 | 2.03 | down |
| ATL8; protein binding / zinc ion binding | AT1G76410 | 2.03 | down |
| hypothetical protein | [AT3G51400](http://www.arabidopsis.org/servlets/TairObject?type=locus&name=AT3G51400) | 2.01 | down |
| hypothetical protein | [AT3G05727](http://www.arabidopsis.org/servlets/TairObject?type=locus&name=AT3G05727) | 2.00 | down |

Genes were identified using the criteria; P<0.05 and fold change >2 or <-2 through One-way ANOVA (with Benjamini Hochberg multiple testing corrections and FDR<0.05) between control and peptide PSY1 treated plants. The up-regulated and down regulated genes were sorted from highest to lowest fold expression.
