# Supplementary material for: Analysis of peptide PSY1 responding transcripts in the two Arabidopsis plant lines: wild type and psy1r receptor mutant
Source: BMC Genomics. 2014 Jun 6;15(1):441. doi: 10.1186/1471-2164-15-441 (PMC4070568; doi:10.1186/1471-2164-15-441)
Supplement: Supplementary file 2 — Additional file 2: Table S2: List of all genes differentially expressed in psy1r mutant plant compared to wild type plants. Genes were identified using the criteria; P < 0.05 and fold change >2 or < -2 through One-way ANOVA (with Benjamini Hochberg multiple testing corrections and FDR < 0.05) between psy1r mutant plants and wild type plants. The up-regulated and down regulated genes were sorted from highest to lowest fold expression values. (DOCX 112 KB) [file 12864_2013_6150_MOESM2_ESM.docx]

**Supplementary table 2: List of all genes differentially expressed in *psy1r* mutant plant compared to wild type plants**

| **Gene Name** | **Gene locus** | **Fold change** | **Regulation** |
| --- | --- | --- | --- |
| ATEXPA2 (ARABIDOPSIS THALIANA EXPANSIN A2) | AT5G05290 | 31.17 | up |
| late embryogenesis abundant protein | [AT1G32560](http://www.arabidopsis.org/servlets/TairObject?type=locus&name=AT1G32560) | 22.70 | up |
| Late embryogenesis abundant protein | [AT4G36600](http://www.arabidopsis.org/servlets/TairObject?type=locus&name=AT4G36600) | 22.29 | up |
| ethylene insensitive 3 family protein | AT5G10120 | 17.01 | up |
| tRNA synthetase-related / tRNA ligase-related | [AT1G18130](http://www.arabidopsis.org/servlets/TairObject?type=locus&name=AT1G18130) | 9.39 | up |
| protein kinase, putative" | AT4G17660 | 6.74 | up |
| CRK6 (CYSTEINE-RICH RLK 6) | [AT4G23140](http://www.arabidopsis.org/servlets/TairObject?type=locus&name=AT4G23140) | 6.70 | up |
| glycosyl hydrolase family 18 protein | [AT4G19750](http://www.arabidopsis.org/servlets/TairObject?type=locus&name=AT4G19750) | 6.57 | up |
| CYP86C1 | [AT1G24540](http://www.arabidopsis.org/servlets/TairObject?type=locus&name=AT1G24540) | 5.23 | up |
| heavy-metal-associated domain-containing protein | [AT5G52710](http://www.arabidopsis.org/servlets/TairObject?type=locus&name=AT5G52710) | 4.86 | up |
| Arabidopsis thaliana PLATZ transcription factor family protein | [AT1G43000](http://www.arabidopsis.org/servlets/TairObject?type=locus&name=AT1G43000) | 4.56 | up |
| hypothetical protein | [AT3G19920](http://www.arabidopsis.org/servlets/TairObject?type=locus&name=AT3G19920) | 4.14 | up |
| transcription factor | [AT3G12910](http://www.arabidopsis.org/servlets/TairObject?type=locus&name=AT3G12910) | 3.97 | up |
| MPC (MATERNALLY EXPRESSED PAB C-TERMINAL) | [AT3G19350](http://www.arabidopsis.org/servlets/TairObject?type=locus&name=AT3G19350) | 3.87 | up |
| PDF1.3 (plant defensin 1.3) | [AT2G26010](http://www.arabidopsis.org/servlets/TairObject?type=locus&name=AT2G26010) | 3.65 | up |
| SEP3 (SEPALLATA3) | [AT1G24260](http://www.arabidopsis.org/servlets/TairObject?type=locus&name=AT1G24260) | 3.64 | up |
| CYP82C2 | [AT4G31970](http://www.arabidopsis.org/servlets/TairObject?type=locus&name=AT4G31970) | 3.58 | up |
| COPT2 | [AT3G46900](http://www.arabidopsis.org/servlets/TairObject?type=locus&name=AT3G46900) | 3.49 | up |
| threonyl-tRNA synthetase, putative / threonine--tRNA ligase, putative" | AT1G17960 | 3.47 | up |
| PDF1.2c (plant defensin 1.2c) | [AT5G44430](http://www.arabidopsis.org/servlets/TairObject?type=locus&name=AT5G44430) | 3.36 | up |
| glycosyl hydrolase family 38 protein | [AT5G66150](http://www.arabidopsis.org/servlets/TairObject?type=locus&name=AT5G66150) | 3.28 | up |
| ELIP2 (EARLY LIGHT-INDUCIBLE PROTEIN 2) | [AT4G14690](http://www.arabidopsis.org/servlets/TairObject?type=locus&name=AT4G14690) | 3.24 | up |
| hypothetical protein | [AT1G35820](http://www.arabidopsis.org/servlets/TairObject?type=locus&name=AT1G35820) | 3.22 | up |
| ATRL4 (ARABIDOPSIS RAD-LIKE 4) | [AT2G18328](http://www.arabidopsis.org/servlets/TairObject?type=locus&name=AT2G18328) | 3.21 | up |
| hypothetical protein | AT2G04515 | 3.11 | up |
| SEP3 (SEPALLATA3) | [AT1G24260](http://arabidopsis.org/servlets/TairObject?type=locus&name=AT1G24260) | 3.09 | up |
| invertase/pectin methylesterase inhibitor family protein | [AT1G23205](http://www.arabidopsis.org/servlets/TairObject?type=locus&name=AT1G23205) | 3.08 | up |
| LECRKA4.1 (LECTIN RECEPTOR KINASE A4.1) | [AT5G01540](http://www.arabidopsis.org/servlets/TairObject?type=locus&name=AT5G01540) | 2.96 | up |
| CYP96A4 | [AT5G52320](http://www.arabidopsis.org/servlets/TairObject?type=locus&name=AT5G52320) | 2.93 | up |
| VQ motif-containing protein | [AT1G78410](http://www.arabidopsis.org/servlets/TairObject?type=locus&name=AT1G78410) | 2.57 | up |
| cinnamoyl-CoA reductase-related | [AT5G14700](http://www.arabidopsis.org/servlets/TairObject?type=locus&name=AT5G14700) | 2.55 | up |
| protein kinase family protein | [AT4G00970](http://www.arabidopsis.org/servlets/TairObject?type=locus&name=AT4G00970) | 2.42 | up |
| AAA-type ATPase family protein | [AT3G28580](http://www.arabidopsis.org/servlets/TairObject?type=locus&name=AT3G28580) | 2.36 | up |
| B120 | [AT4G21390](http://www.arabidopsis.org/servlets/TairObject?type=locus&name=AT4G21390) | 2.34 | up |
| ATMRP3 | [AT3G13080](http://www.arabidopsis.org/servlets/TairObject?type=locus&name=AT3G13080) | 2.31 | up |
| hypothetical protein | [AT2G30900](http://www.arabidopsis.org/servlets/TairObject?type=locus&name=AT2G30900) | 2.29 | up |
| CYP81F2 | [AT5G57220](http://www.arabidopsis.org/servlets/TairObject?type=locus&name=AT5G57220) | 2.21 | up |
| protein binding / zinc ion binding | [AT4G15975](http://www.arabidopsis.org/servlets/TairObject?type=locus&name=AT4G15975) | 2.20 | up |
| glycolipid binding / glycolipid transporter | [AT4G39670](http://www.arabidopsis.org/servlets/TairObject?type=locus&name=AT4G39670) | 2.16 | up |
| hypothetical protein | [AT3G13437](http://www.arabidopsis.org/servlets/TairObject?type=locus&name=AT3G13437) | 2.15 | up |
| hypothetical protein | [AT3G09405](http://www.arabidopsis.org/servlets/TairObject?type=locus&name=AT3G09405) | 2.15 | up |
| acyl-(acyl-carrier-protein) desaturase, putative / stearoyl-ACP desaturase, putative" | AT1G43800 | 2.14 | up |
| hypothetical protein | [AT5G02220](http://www.arabidopsis.org/servlets/TairObject?type=locus&name=AT5G02220) | 2.13 | up |
| NHL3 | [AT5G06320](http://www.arabidopsis.org/servlets/TairObject?type=locus&name=AT5G06320) | 2.11 | up |
| protein kinase family protein | [AT5G46080](http://www.arabidopsis.org/servlets/TairObject?type=locus&name=AT5G46080) | 2.09 | up |
| disease resistance protein (TIR-NBS class), putative" | AT3G04210 | 2.09 | up |
| disease resistance protein-related | [AT4G16880](http://www.arabidopsis.org/servlets/TairObject?type=locus&name=AT4G16880) | 2.08 | up |
| leucine-rich repeat protein kinase, putative" | [AT4G16880](http://www.arabidopsis.org/servlets/TairObject?type=locus&name=AT4G16880) | 2.08 | up |
| COPT2 | [AT3G46900](http://www.arabidopsis.org/servlets/TairObject?type=locus&name=AT3G46900) | 2.04 | up |
| ATEXO70H4 (exocyst subunit EXO70 family protein H4) | [AT3G09520](http://www.arabidopsis.org/servlets/TairObject?type=locus&name=AT3G09520) | 2.03 | up |
| hypothetical protein | [AT4G27980](http://www.arabidopsis.org/servlets/TairObject?type=locus&name=AT4G27980) | 2.02 | up |
| ATDR4; drought-repressed 4 protein (DR4) | [AT1G73330](http://www.arabidopsis.org/servlets/TairObject?type=locus&name=AT1G73330) | 2.02 | up |
| leucine-rich repeat transmembrane protein kinase, putative" | AT1G72300 | 50.2 | down |
| auxin-responsive family protein | [AT2G37030](http://www.arabidopsis.org/servlets/TairObject?type=locus&name=AT2G37030) | 22.49 | down |
| AIG1 (AVRRPT2-INDUCED GENE 1); GTP binding | [AT1G33960](http://www.arabidopsis.org/servlets/TairObject?type=locus&name=AT1G33960) | 14.48 | down |
| DNA binding | [AT1G53490](http://www.arabidopsis.org/servlets/TairObject?type=locus&name=AT1G53490) | 13.03 | down |
| HEI10 HOMOLOG OF HUMAN HEI10 ( ENHANCER OF CELL INVASION NO.10) | [AT3G43572](http://www.arabidopsis.org/servlets/TairObject?type=locus&name=AT3G43572) | 12.62 | down |
| FAB1C | [AT1G71010](http://www.arabidopsis.org/servlets/TairObject?type=locus&name=AT1G71010) | 9.99 | down |
| FAD-binding domain-containing protein | [AT5G44440](http://www.arabidopsis.org/servlets/TairObject?type=locus&name=AT5G44440) | 8.43 | down |
| ARABIDOPSIS MTO 1 RESPONDING DOWN 1 | [AT1G53480](http://www.arabidopsis.org/servlets/TairObject?type=locus&name=AT1G53480) | 7.49 | down |
| triacylglycerol lipase | [AT5G24200](http://www.arabidopsis.org/servlets/TairObject?type=locus&name=AT5G24200) | 7.21 | down |
| peroxidase, putative | [AT5G05340](http://www.arabidopsis.org/servlets/TairObject?type=locus&name=AT5G05340) | 6.80 | down |
| ATBCAT-2 | [AT1G10070](http://www.arabidopsis.org/servlets/TairObject?type=locus&name=AT1G10070) | 6.64 | down |
| hydrolase, acting on ester bonds / lipase | [AT1G58520](http://www.arabidopsis.org/servlets/TairObject?type=locus&name=AT1G58520) | 5.38 | down |
| receptor-like protein kinase, putative" | AT3G45860 | 5.14 | down |
| acyl-(acyl-carrier-protein) desaturase | AT3G02620 | 5.12 | down |
| heavy-metal-associated domain-containing protein | [AT5G26690](http://www.arabidopsis.org/servlets/TairObject?type=locus&name=AT5G26690) | 4.83 | down |
| oxidoreductase, 2OG-Fe(II) oxygenase family protein" | [AT3G55970](http://www.arabidopsis.org/servlets/TairObject?type=locus&name=AT3G55970) | 4.54 | down |
| hypothetical protein | [AT3G08490](http://www.arabidopsis.org/servlets/TairObject?type=locus&name=AT3G08490) | 4.29 | down |
| basic helix-loop-helix (bHLH) family protein | [AT4G20970](http://www.arabidopsis.org/servlets/TairObject?type=locus&name=AT4G20970) | 4.24 | down |
| hypothetical protein | [AT1G29179](http://www.arabidopsis.org/servlets/TairObject?type=locus&name=AT1G29179) | 4.14 | down |
| FAR1 | [AT5G22500](http://www.arabidopsis.org/servlets/TairObject?type=locus&name=AT5G22500) | 4.11 | down |
| QQS (QUA-QUINE STARCH) | [AT3G30720](http://www.arabidopsis.org/servlets/TairObject?type=locus&name=AT3G30720) | 4.08 | down |
| hypothetical protein | [AT5G57760](http://www.arabidopsis.org/servlets/TairObject?type=locus&name=AT5G57760) | 4.02 | down |
| hypothetical protein | [AT5G62280](http://www.arabidopsis.org/servlets/TairObject?type=locus&name=AT5G62280) | 4.02 | down |
| nodulin MtN21 family protein | [AT4G28040](http://www.arabidopsis.org/servlets/TairObject?type=locus&name=AT4G28040) | 3.95 | down |
| hypothetical protein | [AT3G52070](http://www.arabidopsis.org/servlets/TairObject?type=locus&name=AT3G52070) | 3.80 | down |
| OFP16 (ARABIDOPSIS THALIANA OVATE FAMILY PROTEIN 16) | [AT2G32100](http://www.arabidopsis.org/servlets/TairObject?type=locus&name=AT2G32100) | 3.60 | down |
| ICS2 (ISOCHORISMATE SYNTHASE 2) | [AT1G18870](http://www.arabidopsis.org/servlets/TairObject?type=locus&name=AT1G18870) | 3.59 | down |
| UDP-glucoronosyl/UDP-glucosyl transferase family protein | [AT1G01390](http://www.arabidopsis.org/servlets/TairObject?type=locus&name=AT1G01390) | 3.30 | down |
| copper-binding family protein | [AT3G48970](http://www.arabidopsis.org/servlets/TairObject?type=locus&name=AT3G48970) | 3.29 | down |
| U-box domain-containing protein | [AT1G01680](http://www.arabidopsis.org/servlets/TairObject?type=locus&name=AT1G01680) | 3.29 | down |
| MTN3 (Arabidopsis homolog of Medicago truncatula MTN3) | [AT5G23660](http://www.arabidopsis.org/servlets/TairObject?type=locus&name=AT5G23660) | 3.29 | down |
| ATMGL | [AT1G64660](http://www.arabidopsis.org/servlets/TairObject?type=locus&name=AT1G64660) | 3.26 | down |
| GRP3S (GLYCINE-RICH PROTEIN 3 SHORT ISOFORM) | [AT2G05380](http://www.arabidopsis.org/servlets/TairObject?type=locus&name=AT2G05380) | 3.25 | down |
| COR414-TM1 | [AT1G29395](http://www.arabidopsis.org/servlets/TairObject?type=locus&name=AT1G29395) | 3.18 | down |
| hypothetical protein | AT5G21910 | 3.15 | down |
| AtGolS6 | [AT4G26250](http://www.arabidopsis.org/servlets/TairObject?type=locus&name=AT4G26250) | 3.12 | down |
| HEC1 (HECATE 1); transcription factor | [AT5G67060](http://www.arabidopsis.org/servlets/TairObject?type=locus&name=AT5G67060) | 3.11 | down |
| meprin and TRAF homology domain-containing protein | [AT2G05400](http://www.arabidopsis.org/servlets/TairObject?type=locus&name=AT2G05400) | 3.11 | down |
| ATL8; protein binding / zinc ion binding | [AT1G76410](http://www.arabidopsis.org/servlets/TairObject?type=locus&name=AT1G76410) | 3.11 | down |
| FRA8 (FRAGILE FIBER 8); glucuronosyltransferase/ transferase | [AT2G28110](http://www.arabidopsis.org/servlets/TairObject?type=locus&name=AT2G28110) | 3.07 | down |
| INT2 (INOSITOL TRANSPORTER 2) | [AT1G30220](http://www.arabidopsis.org/servlets/TairObject?type=locus&name=AT1G30220) | 3.07 | down |
| hypothetical protein | [AT2G16050](http://www.arabidopsis.org/servlets/TairObject?type=locus&name=AT2G16050) | 3.07 | down |
| DMT2 | [AT4G14140](http://www.arabidopsis.org/servlets/TairObject?type=locus&name=AT4G14140) | 2.97 | down |
| Expressed protein | [AT1G48325](http://www.arabidopsis.org/servlets/TairObject?type=locus&name=AT1G48325) | 2.90 | down |
| ACD6 (ACCELERATED CELL DEATH 6); protein binding | [AT4G14400](http://www.arabidopsis.org/servlets/TairObject?type=locus&name=AT4G14400) | 2.87 | down |
| zinc finger (CCCH-type) family protein | [AT5G44260](http://www.arabidopsis.org/servlets/TairObject?type=locus&name=AT5G44260) | 2.84 | down |
| hypothetical protein | [AT4G16000](http://www.arabidopsis.org/servlets/TairObject?type=locus&name=AT4G16000) | 2.83 | down |
| binding / zinc ion binding | [AT2G26695](http://www.arabidopsis.org/servlets/TairObject?type=locus&name=AT2G26695) | 2.77 | down |
| thiol methyltransferase, putative | [AT2G43920](http://www.arabidopsis.org/servlets/TairObject?type=locus&name=AT2G43920) | 2.75 | down |
| Arabidopsis thaliana 12-oxophytodienoate reductase | AT1G09400 | 2.68 | down |
| hypothetical protein | [AT3G62990](http://www.arabidopsis.org/servlets/TairObject?type=locus&name=AT3G62990) | 2.63 | down |
| HB-2 (HOMEOBOX-2); DNA binding / transcription factor | [AT2G18550](http://www.arabidopsis.org/servlets/TairObject?type=locus&name=AT2G18550) | 2.63 | down |
| SEN1 (SENESCENCE 1) | [AT4G35770](http://www.arabidopsis.org/servlets/TairObject?type=locus&name=AT4G35770) | 2.62 | down |
| misc_RNA | [AT3G27884](http://www.arabidopsis.org/servlets/TairObject?type=locus&name=AT3G27884) | 2.60 | down |
| auxin-responsive protein-related | [AT5G20820](http://www.arabidopsis.org/servlets/TairObject?type=locus&name=AT5G20820) | 2.59 | down |
| M10 | [AT2G41280](http://www.arabidopsis.org/servlets/TairObject?type=locus&name=AT2G41280) | 2.59 | down |
| FRU (FER-LIKE REGULATOR OF IRON UPTAKE) | [AT2G28160](http://www.arabidopsis.org/servlets/TairObject?type=locus&name=AT2G28160) | 2.58 | down |
| tetracycline transporter | [AT2G16990](http://www.arabidopsis.org/servlets/TairObject?type=locus&name=AT2G16990) | 2.55 | down |
| NGA4 (NGATHA4); transcription factor | [AT4G01500](http://www.arabidopsis.org/servlets/TairObject?type=locus&name=AT4G01500) | 2.54 | down |
| HEC2 (HECATE 2); DNA binding / transcription factor | [AT3G50330](http://www.arabidopsis.org/servlets/TairObject?type=locus&name=AT3G50330) | 2.53 | down |
| hypothetical protein | [AT5G46874](http://www.arabidopsis.org/servlets/TairObject?type=locus&name=AT5G46874) | 2.52 | down |
| protein kinase family protein | [AT5G57670](http://www.arabidopsis.org/servlets/TairObject?type=locus&name=AT5G57670) | 2.45 | down |
| PUP1 (PURINE PERMEASE 1) | [AT1G28230](http://www.arabidopsis.org/servlets/TairObject?type=locus&name=AT1G28230) | 2.44 | down |
| SNZ (SCHNARCHZAPFEN); DNA binding / transcription factor | [AT2G39250](http://www.arabidopsis.org/servlets/TairObject?type=locus&name=AT2G39250) | 2.44 | down |
| hypothetical protein | [AT1G53870](http://www.arabidopsis.org/servlets/TairObject?type=locus&name=AT1G53870) | 2.41 | down |
| 2-oxoisovalerate dehydrogenase, putative | AT1G21400 | 2.39 | down |
| S-locus protein kinase, putative" | AT1G61480 | 2.39 | down |
| hypothetical protein | [AT3G10120](http://www.arabidopsis.org/servlets/TairObject?type=locus&name=AT3G10120) | 2.37 | down |
| cytochrome P450 family protein | [AT3G44970](http://www.arabidopsis.org/servlets/TairObject?type=locus&name=AT3G44970) | 2.37 | down |
| short-chain dehydrogenase/reductase (SDR) family protein | [AT5G02540](http://www.arabidopsis.org/servlets/TairObject?type=locus&name=AT5G02540) | 2.36 | down |
| ORP4C | [AT5G57240](http://www.arabidopsis.org/servlets/TairObject?type=locus&name=AT5G57240) | 2.36 | down |
| UGT72E1 (UDP-glucosyl transferase 72E1) | [AT3G50740](http://www.arabidopsis.org/servlets/TairObject?type=locus&name=AT3G50740) | 2.36 | down |
| GA3OX1 (GIBBERELLIN 3-OXIDASE 1) | [AT1G15550](http://www.arabidopsis.org/servlets/TairObject?type=locus&name=AT1G15550) | 2.33 | down |
| 3-beta-hydroxy-delta5-steroid dehydrogenase | [AT1G76470](http://www.arabidopsis.org/servlets/TairObject?type=locus&name=AT1G76470) | 2.32 | down |
| hypothetical protein | [AT2G35585](http://www.arabidopsis.org/servlets/TairObject?type=locus&name=AT2G35585) | 2.31 | down |
| PI (PISTILLATA); DNA binding / transcription factor | [AT5G20240](http://www.arabidopsis.org/servlets/TairObject?type=locus&name=AT5G20240) | 2.31 | down |
| PDF1.5 (plant defensin 1.5) | [AT1G55010](http://www.arabidopsis.org/servlets/TairObject?type=locus&name=AT1G55010) | 2.28 | down |
| hypothetical protein | [AT2G07795](http://www.arabidopsis.org/servlets/TairObject?type=locus&name=AT2G07795) | 2.28 | down |
| KNATM (KNOX ARABIDOPSIS THALIANA MEINOX) | [AT1G14760](http://www.arabidopsis.org/servlets/TairObject?type=locus&name=AT1G14760) | 2.28 | down |
| hydrolase | [AT2G03550](http://www.arabidopsis.org/servlets/TairObject?type=locus&name=AT2G03550) | 2.23 | down |
| protein phosphatase 2C family protein / PP2C family protein | [AT5G02760](http://www.arabidopsis.org/servlets/TairObject?type=locus&name=AT5G02760) | 2.22 | down |
| ORP4C (OSBP(OXYSTEROL BINDING PROTEIN)-RELATED PROTEIN 4C) | [AT5G57240](http://www.arabidopsis.org/servlets/TairObject?type=locus&name=AT5G57240) | 2.21 | down |
| PCK2 (PHOSPHOENOLPYRUVATE CARBOXYKINASE 2) | [AT5G65690](http://www.arabidopsis.org/servlets/TairObject?type=locus&name=AT5G65690) | 2.20 | down |
| ethylene-responsive factor, putative" | AT4G18450 | 2.20 | down |
| GRP3S (GLYCINE-RICH PROTEIN 3 SHORT ISOFORM) | [AT2G05380](http://www.arabidopsis.org/servlets/TairObject?type=locus&name=AT2G05380) | 2.16 | down |
| zinc finger (C3HC4-type RING finger) family protein | [AT4G28270](http://www.arabidopsis.org/servlets/TairObject?type=locus&name=AT4G28270) | 2.15 | down |
| zinc finger (GATA type) family protein | [AT3G60530](http://www.arabidopsis.org/servlets/TairObject?type=locus&name=AT3G60530) | 2.14 | down |
| OFP15 (ARABIDOPSIS THALIANA OVATE FAMILY PROTEIN 15) | [AT2G36050](http://www.arabidopsis.org/servlets/TairObject?type=locus&name=AT2G36050) | 2.14 | down |
| zinc finger (GATA type) family protein | [AT3G60530](http://www.arabidopsis.org/servlets/TairObject?type=locus&name=AT3G60530) | 2.13 | down |
| SAP domain-containing protein | [AT5G66840](http://www.arabidopsis.org/servlets/TairObject?type=locus&name=AT5G66840) | 2.12 | down |
| hypothetical protein | [AT1G13670](http://www.arabidopsis.org/servlets/TairObject?type=locus&name=AT1G13670) | 2.12 | down |
| PWD (PHOSPHOGLUCAN, WATER DIKINASE) | [AT4G24450](http://www.arabidopsis.org/servlets/TairObject?type=locus&name=AT4G24450) | 2.11 | down |
| hypothetical protein | [AT3G51400](http://www.arabidopsis.org/servlets/TairObject?type=locus&name=AT3G51400) | 2.10 | down |
| hypothetical protein | [AT3G05727](http://www.arabidopsis.org/servlets/TairObject?type=locus&name=AT3G05727) | 2.10 | down |
| CYP96A9 (CYTOCHROME P450 96 A9) | [AT4G39480](http://www.arabidopsis.org/servlets/TairObject?type=locus&name=AT4G39480) | 2.10 | down |
| FCA; RNA binding | [AT4G16280](http://www.arabidopsis.org/servlets/TairObject?type=locus&name=AT4G16280) | 2.09 | down |
| protease inhibitor/seed storage/lipid transfer protein (LTP) family protein | [AT4G22520](http://www.arabidopsis.org/servlets/TairObject?type=locus&name=AT4G22520) | 2.07 | down |
| TSA1 (TSK-ASSOCIATING PROTEIN 1) | [AT1G52410](http://www.arabidopsis.org/servlets/TairObject?type=locus&name=AT1G52410) | 2.07 | down |
| zinc finger (C3HC4-type RING finger) family protein | [AT3G58720](http://www.arabidopsis.org/servlets/TairObject?type=locus&name=AT3G58720) | 2.04 | down |
| hypothetical protein | [AT3G62990](http://www.arabidopsis.org/servlets/TairObject?type=locus&name=AT3G62990) | 2.02 | down |
| LNG1 (LONGIFOLIA1) | AT2G11240 | 2.01 | down |
| octicosapeptide/Phox/Bem1p (PB1) domain-containing protein | [AT3G26510](http://www.arabidopsis.org/servlets/TairObject?type=locus&name=AT3G26510) | 2.00 | down |

Genes were identified using the criteria; P<0.05 and fold change >2 or <-2 through One-way ANOVA (with Benjamini Hochberg multiple testing corrections and FDR<0.05) between *psy1r* mutant plants and wild type plants. The up-regulated and down regulated genes were sorted from highest to lowest fold expression.
