# Supplementary material for: Analysis of peptide PSY1 responding transcripts in the two Arabidopsis plant lines: wild type and psy1r receptor mutant
Source: BMC Genomics. 2014 Jun 6;15(1):441. doi: 10.1186/1471-2164-15-441 (PMC4070568; doi:10.1186/1471-2164-15-441)
Supplement: Supplementary file 3 — Additional file 3: Table S3: List of genes differentially expressed in PSY1-treated psy1r mutant plants compared to peptide treated wild type plants. Genes were identified using the criteria; P < 0.05 and fold change >2 or < -2 through One-way ANOVA (with Benjamini Hochberg multiple testing corrections and FDR < 0.05) between PSY1 treated psy1r mutant plants and PSY1 treated-wild type plants. The up-regulated and down regulated genes were sorted from highest to lowest fold expression values. (DOCX 120 KB) [file 12864_2013_6150_MOESM3_ESM.docx]

**Supplementary table 3: List of genes differentially expressed in PSY1-treated *psy1r* mutant plants compared to peptide treated wild type plants**

| **Gene Name** | **Gene locus** | **Fold change** | **Regulation** |
| --- | --- | --- | --- |
| hypothetical protein | AT1G33940 | 37.24 | up |
| aspartyl protease family protein | AT5G45120 | 31.63 | up |
| IRT1 (iron-regulated transporter 1) | AT4G19690 | 18.03 | up |
| hypothetical protein | AT4G09210 | 17.29 | up |
| MPC | AT3G19350 | 15.24 | up |
| hypothetical protein | AT3G19920 | 11.68 | up |
| CYP82C2 | AT4G31970 | 10.38 | up |
| late embryogenesis abundant group 1 domain-containing protein | AT1G32560 | 10.24 | up |
| glycosyl hydrolase family 18 protein | AT4G19750 | 9.68 | up |
| hypothetical protein | AT1G10100 | 9.62 | up |
| hypothetical protein | AT4G36600 | 7.73 | up |
| pectinesterase family protein | AT5G20860 | 7.30 | up |
| ethylene insensitive 3 family protein | AT5G10120 | 6.79 | up |
| transcription factor | AT1G10585 | 6.63 | up |
| hypothetical protein | AT2G41650 | 5.71 | up |
| ICL (ISOCITRATE LYASE) | AT3G21720 | 5.40 | up |
| tRNA synthetase-related / tRNA ligase-related | AT1G18130 | 5.35 | up |
| basic helix-loop-helix (bHLH) family protein | AT4G20970 | 5.34 | up |
| protein kinase, putative" | AT4G17660 | 5.31 | up |
| NADP-dependent oxidoreductase, putative" | AT5G16960 | 5.23 | up |
| CRK6 (CYSTEINE-RICH RLK 6); kinase | AT4G23140 | 5.14 | up |
| hypothetical protein | AT1G16480 | 4.61 | up |
| transducin family protein / WD-40 repeat family protein | AT3G18950 | 4.59 | up |
| ATEXPA2 | AT5G05290 | 4.36 | up |
| f-box family protein | AT2G27310 | 4.24 | up |
| zinc-binding family protein | AT1G43000 | 4.00 | up |
| hypothetical protein | AT3G62990 | 3.94 | up |
| LOX3; electron carrier/ iron ion binding | AT1G17420 | 3.90 | up |
| plastocyanin-like domain-containing protein | AT5G07475 | 3.72 | up |
| MD-2-related lipid recognition domain-containing protein | AT2G16005 | 3.57 | up |
| MLO12 | AT2G39200 | 3.40 | up |
| CYP96A4 | AT5G52320 | 3.29 | up |
| MYB112 | AT1G48000 | 3.27 | up |
| ATDR4 | AT1G73330 | 3.15 | up |
| MYB7 | AT2G16720 | 3.12 | up |
| AAA-type ATPase family protein | AT3G28580 | 3.11 | up |
| CYP72A13; electron carrier | AT3G14660 | 3.08 | up |
| MYB15 | AT3G23250 | 3.03 | up |
| glycosyl hydrolase family 38 protein | AT5G66150 | 3.03 | up |
| AtRLP22 | AT2G32660 | 3.01 | up |
| hypothetical protein | AT2G41650 | 2.91 | up |
| hypothetical protein | AT4G36600 | 2.86 | up |
| SVL3 (SHV3-LIKE 3) | AT3G20520 | 2.84 | up |
| VQ motif-containing protein | AT1G78410 | 2.84 | up |
| hypothetical protein | AT3G62990 | 2.79 | up |
| SEP3 (SEPALLATA3) | AT1G24260 | 2.76 | up |
| cinnamoyl-CoA reductase-related | AT5G14700 | 2.70 | up |
| AtMYB10 | AT3G24210 | 2.69 | up |
| GDSL-motif lipase/hydrolase family protein | AT5G03610 | 2.69 | up |
| glycolipid binding / glycolipid transporter | AT4G39670 | 2.68 | up |
| LOX3 | AT1G17420 | 2.58 | up |
| protein kinase family protein | AT5G46080 | 2.58 | up |
| threonyl-tRNA synthetase | AT1G17960 | 2.56 | up |
| transcription factor | AT3G12910 | 2.56 | up |
| DNA-binding protein-related | AT2G38250 | 2.46 | up |
| ATRL4 (ARABIDOPSIS RAD-LIKE 4) | AT2G18328 | 2.45 | up |
| aspartyl protease family protein | AT2G39710 | 2.41 | up |
| WRKY6 | AT1G62300 | 2.41 | up |
| ATTI1 | AT2G43510 | 2.38 | up |
| disease resistance-responsive protein-related | AT2G21100 | 2.35 | up |
| CTP synthase/ catalytic | AT4G20320 | 2.33 | up |
| ATMPK11 | AT1G01560 | 2.32 | up |
| ATEXPA16 (ARABIDOPSIS THALIANA EXPANSIN A16) | AT3G55500 | 2.30 | up |
| ELIP2 (EARLY LIGHT-INDUCIBLE PROTEIN 2) | AT4G14690 | 2.29 | up |
| cysteine proteinase, putative" | AT2G27420 | 2.28 | up |
| oxidoreductase, 2OG-Fe(II) oxygenase family protein" | AT3G55970 | 2.27 | up |
| Expressed protein | AT4G28405 | 2.27 | up |
| hypothetical protein | AT5G59790 | 2.22 | up |
| protein kinase family protein | AT4G00970 | 2.21 | up |
| calcium-binding protein, putative | AT4G20780 | 2.19 | up |
| peroxidase, putative" | AT5G05340 | 2.19 | up |
| leucine-rich repeat protein kinase, putative" | AT5G56040 | 2.17 | up |
| hypothetical protein | AT1G49000 | 2.17 | up |
| ankyrin protein kinase, putative" | AT4G18950 | 2.16 | up |
| hypothetical protein | AT1G20310 | 2.15 | up |
| curculin-like (mannose-binding) lectin family protein | AT1G78860 | 2.13 | up |
| MATE efflux family protein | AT1G73700 | 2.12 | up |
| disease resistance protein (TIR-NBS-LRR class), putative" | AT3G04210 | 2.10 | up |
| COPT2 | AT3G46900 | 2.08 | up |
| proton-dependent oligopeptide transport (POT) family protein | AT3G47960 | 2.07 | up |
| hypothetical protein | AT4G16447 | 2.07 | up |
| cell expansion protein, putative | AT5G49680 | 2.04 | up |
| cation efflux family protein / metal tolerance protein, putative (MTPc3) | AT3G58060 | 2.04 | up |
| auxin-responsive GH3 family protein | AT1G48670 | 2.01 | up |
| CKX2 (CYTOKININ OXIDASE 2) | AT1G47620 | 2.01 | up |
| CYP96A8 | AT3G50610 | 2.01 | up |
| unknown protein | AT4G26880 | 2.01 | up |
| stigma-specific Stig1 family protein | AT2G13335 | 2.00 | up |
| hypothetical protein | AT3G43572 | 47.71 | down |
| leucine-rich repeat transmembrane protein kinase, putative" | AT1G72300 | 45.96 | down |
| auxin-responsive family protein | AT2G37030 | 17.22 | down |
| SCRL3 (SCR-Like 3) | AT1G08695 | 12.41 | down |
| hypothetical protein | AT5G35870 | 9.80 | down |
| DNA binding | AT1G53490 | 8.40 | down |
| QQS (QUA-QUINE STARCH) | AT3G30720 | 7.77 | down |
| FAB1C | AT1G71010 | 7.35 | down |
| heat shock protein binding | AT1G71000 | 6.60 | down |
| hypothetical protein | AT1G53480 | 6.27 | down |
| hypothetical protein | AT5G57760 | 6.23 | down |
| SCRL17 (SCR-Like 17) | AT2G25685 | 5.83 | down |
| FAD-binding domain-containing protein | AT5G44440 | 5.83 | down |
| hypothetical protein | AT5G57760 | 5.70 | down |
| hypothetical protein | AT4G36230 | 5.68 | down |
| misc_RNA | AT1G66173 | 5.37 | down |
| zinc finger (C3HC4-type RING finger) family protein | AT5G47610 | 4.76 | down |
| F-box family protein-related | AT1G64295 | 4.53 | down |
| SRO4 (SIMILAR TO RCD ONE 4); NAD+ ADP-ribosyltransferase | AT3G47720 | 4.46 | down |
| FAR1 (FATTY ACID REDUCTASE 1) | AT5G22500 | 4.43 | down |
| hypothetical protein | AT1G29179 | 4.34 | down |
| WVD2 | AT5G28646 | 4.24 | down |
| ATBCAT-2 | AT1G10070 | 3.94 | down |
| receptor-like protein kinase, putative" | AT3G45860 | 3.82 | down |
| hypothetical protein | AT2G32785 | 3.78 | down |
| hypothetical protein | AT1G68170 | 3.77 | down |
| nodulin MtN21 family protein | AT4G01600 | 3.72 | down |
| GRAM domain-containing protein | AT1G13609 | 3.70 | down |
| hypothetical protein | AT1G01390 | 3.52 | down |
| UDP-glucoronosyl | AT4G35770 | 3.31 | down |
| SEN1 (SENESCENCE 1) | AT1G43800 | 3.23 | down |
| acyl-(acyl-carrier-protein) desaturase, putative | AT5G08460 | 3.23 | down |
| GDSL-motif lipase/hydrolase family protein | AT2G36050 | 3.20 | down |
| OFP15 (ARABIDOPSIS THALIANA OVATE FAMILY PROTEIN 15) | AT5G23660 | 3.16 | down |
| MTN3 (Arabidopsis homolog of Medicago truncatula MTN3) | AT4G30380 | 3.05 | down |
| EXLB2 (EXPANSIN-LIKE B2 PRECURSOR) | AT2G41230 | 3.03 | down |
| hypothetical protein | AT1G67860 | 3.01 | down |
| hypothetical protein | AT3G55240 | 2.99 | down |
| hypothetical protein | AT5G46874 | 2.98 | down |
| hypothetical protein | AT4G30420 | 2.97 | down |
| nodulin MtN21 family protein | AT2G05380 | 2.95 | down |
| GRP3S (GLYCINE-RICH PROTEIN 3 SHORT ISOFORM) | AT4G22520 | 2.89 | down |
| protease inhibitor/seed storage/lipid transfer protein (LTP) family protein | AT3G51220 | 2.87 | down |
| hypothetical protein | AT4G28040 | 2.86 | down |
| nodulin MtN21 family protein | AT5G03120 | 2.84 | down |
| hypothetical protein | AT5G44650 | 2.83 | down |
| hypothetical protein | AT4G28040 | 2.83 | down |
| nodulin MtN21 family protein | AT4G14140 | 2.78 | down |
| DMT2 (DNA METHYLTRANSFERASE 2) | AT3G07310 | 2.78 | down |
| hypothetical protein | AT1G68568 | 2.75 | down |
| misc_RNA | AT3G05727 | 2.72 | down |
| hypothetical protein | AT3G27050 | 2.71 | down |
| hypothetical protein | AT4G01600 | 2.69 | down |
| DYL1 (DORMANCY-ASSOCIATED PROTEIN-LIKE 1) | AT1G28330 | 2.69 | down |
| AIG1 (AVRRPT2-INDUCED GENE 1); GTP binding | AT3G54920 | 2.67 | down |
| PMR6 (powdery mildew resistant 6); lyase/ pectate lyase | AT1G55010 | 2.66 | down |
| PDF1.5 (plant defensin 1.5) | AT1G70985 | 2.65 | down |
| hydroxyproline-rich glycoprotein family protein | AT2G32100 | 2.64 | down |
| OFP16 (ARABIDOPSIS THALIANA OVATE FAMILY PROTEIN 16) | AT1G73040 | 2.63 | down |
| jacalin lectin family protein | AT3G04030 | 2.62 | down |
| myb family transcription factor | AT5G44260 | 2.62 | down |
| zinc finger (CCCH-type) family protein | AT2G26080 | 2.59 | down |
| AtGLDP2 (Arabidopsis thaliana glycine decarboxylase P-protein 2) | AT1G44414 | 2.59 | down |
| hypothetical protein | AT5G20250 | 2.57 | down |
| DIN10 (DARK INDUCIBLE 10); hydrolase, hydrolyzing O-glycosyl compounds | AT5G20240 | 2.56 | down |
| PI (PISTILLATA); DNA binding / transcription factor | AT5G02540 | 2.55 | down |
| short-chain dehydrogenase/reductase (SDR) family protein | AT2G27290 | 2.55 | down |
| hypothetical protein | AT5G18240 | 2.53 | down |
| MYR1 (MYb-related protein 1); transcription factor | AT1G68320 | 2.52 | down |
| MYB62 (myb domain protein 62); DNA binding / transcription factor | AT1G76470 | 2.52 | down |
| 3-beta-hydroxy-delta5-steroid dehydrogenase | AT4G28030 | 2.51 | down |
| GCN5-related N-acetyltransferase (GNAT) family protein | AT5G25980 | 2.50 | down |
| TGG2 (GLUCOSIDE GLUCOHYDROLASE 2) | AT4G39480 | 2.48 | down |
| CYP96A9 (CYTOCHROME P450 96 A9) | AT3G48970 | 2.47 | down |
| copper-binding family protein | AT3G51220 | 2.45 | down |
| hypothetical protein | AT2G42890 | 2.44 | down |
| AML2 (ARABIDOPSIS-MEI2-LIKE 2) | AT4G16000 | 2.44 | down |
| hypothetical protein | AT3G58720 | 2.43 | down |
| zinc finger (C3HC4-type RING finger) family protein | AT4G16070 | 2.40 | down |
| lipase class 3 family protein | AT1G06080 | 2.38 | down |
| ADS1 (DELTA 9 DESATURASE 1); oxidoreductase | AT1G09400 | 2.36 | down |
| 12-oxophytodienoate reductase, putative" | AT2G38320 | 2.35 | down |
| hypothetical protein | AT4G38530 | 2.35 | down |
| ATPLC1 (ARABIDOPSIS THALIANA PHOSPHOLIPASE C 1) | AT4G35770 | 2.35 | down |
| SEN1 (SENESCENCE 1) | AT2G04420 | 2.33 | down |
| nucleic acid binding | AT5G62280 | 2.33 | down |
| hypothetical protein | AT3G50330 | 2.32 | down |
| HEC2 (HECATE 2) | AT5G15720 | 2.32 | down |
| GLIP7; carboxylesterase/ lipase | AT5G26690 | 2.32 | down |
| heavy-metal-associated domain-containing protein | AT4G07740 | 2.31 | down |
| hypothetical protein | AT5G44562 | 2.31 | down |
| misc_RNA | AT2G26695 | 2.31 | down |
| binding / zinc ion binding | AT3G50740 | 2.29 | down |
| UGT72E1 (UDP-glucosyl transferase 72E1) | AT1G35820 | 2.28 | down |
| hypothetical protein | AT5G18130 | 2.28 | down |
| hypothetical protein | AT2G05380 | 2.26 | down |
| GRP3S (GLYCINE-RICH PROTEIN 3 SHORT ISOFORM) | AT3G16190 | 2.26 | down |
| isochorismatase hydrolase family protein | AT4G01500 | 2.26 | down |
| NGA4 (NGATHA4); transcription factor | AT2G16050 | 2.25 | down |
| hypothetical protein | AT3G53530 | 2.25 | down |
| heavy-metal-associated domain-containing protein | AT2G34170 | 2.24 | down |
| hypothetical protein | AT1G30220 | 2.23 | down |
| INT2 (INOSITOL TRANSPORTER 2) | AT1G28230 | 2.22 | down |
| PUP1 (PURINE PERMEASE 1) | AT3G26280 | 2.20 | down |
| CYP71B4 | AT5G16540 | 2.20 | down |
| ZFN3 (ZINC FINGER NUCLEASE 3) | AT4G31248 | 2.20 | down |
| misc_RNA | AT3G16070 | 2.18 | down |
| hypothetical protein | AT2G02020 | 2.18 | down |
| proton-dependent oligopeptide transport (POT) family protein | AT1G18330 | 2.17 | down |
| EPR1 (EARLY-PHYTOCHROME-RESPONSIVE1) | AT5G21910 | 2.16 | down |
| hypothetical protein | AT1G14760 | 2.16 | down |
| KNATM (KNOX ARABIDOPSIS THALIANA MEINOX) | AT5G02760 | 2.14 | down |
| protein phosphatase 2C family protein / PP2C family protein | AT1G74055 | 2.14 | down |
| hypothetical protein | AT5G27320 | 2.13 | down |
| GID1C (GA INSENSITIVE DWARF1C); hydrolase | AT3G05730 | 2.12 | down |
| hypothetical protein | AT1G14190 | 2.12 | down |
| glucose-methanol-choline (GMC) oxidoreductase family protein | AT5G51020 | 2.11 | down |
| CRL (CRUMPLED LEAF) | AT3G58980 | 2.11 | down |
| F-box family protein | AT1G49130 | 2.09 | down |
| zinc finger (B-box type) family protein | AT5G05430 | 2.09 | down |
| hypothetical protein | AT5G59890 | 2.09 | down |
| ADF4 (ACTIN DEPOLYMERIZING FACTOR 4) | AT3G22886 | 2.08 | down |
| misc_RNA | AT2G05380 | 2.08 | down |
| GRP3S (GLYCINE-RICH PROTEIN 3 SHORT ISOFORM) | AT1G58520 | 2.08 | down |
| hydrolase, acting on ester bonds / lipase | AT3G44970 | 2.07 | down |
| cytochrome P450 family protein | AT2G28160 | 2.06 | down |
| FRU (FER-LIKE REGULATOR OF IRON UPTAKE) | AT2G43920 | 2.06 | down |
| thiol methyltransferase, putative | AT4G24450 | 2.05 | down |
| PWD (PHOSPHOGLUCAN, WATER DIKINASE) | AT3G01550 | 2.04 | down |
| PPT2 (PHOSPHOENOLPYRUVATE (PEP)/PHOSPHATE TRANSLOCATOR 2) | AT1G03055 | 2.04 | down |
| hypothetical protein | AT5G25490 | 2.04 | down |
| zinc finger (Ran-binding) family protein | AT2G01620 | 2.04 | down |
| MEE11 (maternal effect embryo arrest 11) | AT1G73960 | 2.04 | down |
| TAF2 (TBP-ASSOCIATED FACTOR 2) | AT4G33520 | 2.04 | down |
| PAA1 (P-TYPE ATP-ASE 1) | AT2G27030 | 2.03 | down |
| CAM5 (CALMODULIN 5); calcium ion binding | AT2G20920 | 2.02 | down |
| hypothetical protein | AT3G63300 | 2.02 | down |
| phosphoinositide binding | AT5G57240 | 2.02 | down |
| ORP4C (OSBP(OXYSTEROL BINDING PROTEIN)-RELATED PROTEIN 4C) | AT3G26510 | 2.01 | down |
| octicosapeptide/Phox/Bem1p (PB1) domain-containing protein | AT4G17840 | 2.01 | down |
| hypothetical protein | AT1G14700 | 2.01 | down |
| PAP3 (PURPLE ACID PHOSPHATASE 3) | AT3G52070 | 2.01 | down |
| hypothetical protein | AT3G05140 | 2.01 | down |
| RBK2 (Rop Binding protein Kinases 2) | AT1G55310 | 2.01 | down |
| SR33; RNA binding / protein binding | AT5G15580 | 2.01 | down |
| LNG1 (LONGIFOLIA1) | AT2G11240 | 2.01 | down |
| transposable element gene | AT4G40090 | 2.01 | down |
| AGP3 (arabinogalactan-protein 3) | AT3G56590 | 2.01 | down |
| hydroxyproline-rich glycoprotein family protein | AT3G09470 | 2.01 | down |
| unknown protein | AT5G26740 | 2.01 | down |
| protease inhibitor/seed storage/lipid transfer protein (LTP) family protein | AT3G22620 | 2.01 | down |
| protein kinase family protein | AT5G63370 | 2.01 | down |
| MATE efflux family protein | AT3G26590 | 2.01 | down |
| splicing factor PWI domain-containing protein | AT1G60200 | 2.01 | down |
| HMG1 (HYDROXY METHYLGLUTARYL COA REDUCTASE 1) | AT1G76490 | 2.01 | down |
| ARR1 (ARABIDOPSIS RESPONSE REGULATOR 1) | AT3G16857 | 2.01 | down |
| unknown protein | AT1G21830 | 2.01 | down |
| unknown protein | AT5G22310 | 2.00 | down |
| phosphatidylinositol 3- and 4-kinase family protein | AT1G13640 | 2.00 | down |
| PLDGAMMA2; phospholipase D | AT4G11830 | 2.00 | down |
| transferase family protein | AT3G62160 | 2.00 | down |
| IAA19 (INDOLE-3-ACETIC ACID INDUCIBLE 19) | AT3G15540 | 2.00 | down |
| 5'-AMP-activated protein kinase beta-2 subunit, putative | AT5G21170 | 2.00 | down |
| DNA-binding family protein / remorin family protein | AT3G61260 | 2.00 | down |
| transposable element gene | AT2G13890 | 2.00 | down |
| zinc finger (GATA type) family protein | AT2G45050 | 2.00 | down |
| BEE1 (BR Enhanced Expression 1); transcription factor | AT1G18400 | 2.00 | down |
| unknown protein | AT4G31080 | 2.00 | down |
| protein phosphatase 2C family protein / PP2C family protein | AT3G17090 | 2.00 | down |
| unknown protein | AT4G12690 | 2.00 | down |
| glutamine amidotransferase-related | AT1G15040 | 2.00 | down |
| ATHB52 (ARABIDOPSIS THALIANA HOMEOBOX PROTEIN 52) | AT5G53980 | 2.00 | down |
| AWPM-19-like membrane family protein | AT5G18970 | 2.00 | down |

Genes were identified using the criteria; P<0.05 and fold change >2 or <-2 through One-way ANOVA (with Benjamini Hochberg multiple testing corrections and FDR<0.05) between peptide PSY1 treated-*psy1r* mutant plants and PSY1 treated-wild type plants. The up-regulated and down regulated genes were sorted from highest to lowest fold expression.
