# Supplementary material for: Analysis of peptide PSY1 responding transcripts in the two Arabidopsis plant lines: wild type and psy1r receptor mutant
Source: BMC Genomics. 2014 Jun 6;15(1):441. doi: 10.1186/1471-2164-15-441 (PMC4070568; doi:10.1186/1471-2164-15-441)
Supplement: Supplementary file 4 — Additional file 4: Table S4: Functional grouping of genes showing differential transcript expression in different treatments. Gene ontology (GO) enrichment was performed in AgriGO (FDR correction and Fisher’s exact test < 0.05) using the whole Arabidopsis genome as the background/reference. (DOCX 25 KB) [file 12864_2013_6150_MOESM4_ESM.docx]

**Supplementary table 4: Functional grouping of genes showing differential transcript expression in different treatments**

| Gene class | Putative function | *psy1r* vs WT | | | WT+PSY1 vs WT | | |
| --- | --- | --- | --- | --- | --- | --- | --- |
|  |  | No. | % | Examples | No. | % | Examples |
| Cellular and biosynthetic processes | **Cellular processes** | 73 | 46 | ABCC3 ACD6 AIG1 AT1G10585 AT1G17960 AT1G18130 AT1G61480 AT1G76470 AT1G78410 AT2G16990 AT3G02620 AT3G04210 AT3G12910 AT3G25790 AT3G52070 AT4G15975 AT4G17660 AT4G18450 AT4G20970 AT4G39670 AT5G02760 AT5G10120 AT5G14700 AT5G44260 AT5G46080 AT5G57670 B120 BCAT-2 COPT2 COR413IM1 CRK4 CRK41 CRK6 CYP81F2 CYP82C2 DIN2 DMT2 ELIP2 EXO70H4 EXPA2 FAB1C FAR1 FCA FRA8 FRU FTM1 GA3OX1 GATA4 GRP3S HB21 HEC1 HEC2 HEI10 ICL ICS2 INT2 KNATM LECRKA4.1 MGL MPC MYB112 NGA4 OFP15 OFP16 PDF1.2b PI PSY1R PUB54 PWD QQS SEP3 SNZ UGT72E1 AT5G15580 AT5G21910 | 34 | 44 | 5PTASE11 ADF4 AT1G10585 AT2G27290 AT3G07310 AT3G51450 AT4G17250 AT4G18450 AT4G20970 AT4G28030 AT5G08460 AT5G10700 AT5G20860 AT5G55450 AT5G64420 CEST CRL DIN2 DREB2A FKD1 GPX7 ICL ML2 MPC MPK11 MYB112 MYB7 PDF1.2b PPT2 RALFL22 RALFL33 SCRL3 UMAMIT23 WVD2 |
|  | **Biosynthetic process** | 44 | 27 | AIG1 AT1G10585 AT1G17960 AT1G18130 AT1G76470 AT1G78410 AT3G02620 AT3G12910 AT3G25790 AT3G52070 AT4G18450 AT4G20970 AT4G39670 AT5G10120 AT5G14700 AT5G44260 AT5G46080 CYP81F2 ELIP2 FAR1 FCA FRA8 FRU FTM1 GA3OX1 GATA4 GolS6 GRP3S HB21 HEC1 HEC2 ICS2 KNATM MPC MYB112 NGA4 OFP15 OFP16 PCK2 PI QQS SEP3 SNZ UGT72E1 AT5G15580 AT5G21910 | 18 | 23 | AT1G10585 AT2G27290 AT3G51450 AT4G18450 AT4G20970 AT4G28030 AT5G08460 AT5G55450 AT5G64420 DREB2A GolS6 MPC MPK11 MYB112 MYB7 PPT2 RALFL22 UMAMIT23 |
| Regulation | **Cell communication and signal transduction** | 16 | 10 | AIG1, AT1G61480, AT1G78410, AT3G04210, AT4G15975, AT4G18450, AT4G39670, AT5G10120, B120, COR413IM1, FTM1, GA3OX1, LECRKA4.1, MGL, PDF1.2b, PSY1R | 11 | 14 | ADF4 AT3G07310 AT4G18450 AT5G55450 FKD1 MPK11 MYB7 PDF1.2b RALFL22 RALFL33 SCRL3 |
|  | **Protein kinase/phosphatases** | 12 | 8 | AT1G61480, AT4G17660, AT5G46080, AT5G57670, B120, CRK4, CRK41, CRK6, FAB1C, LECRKA4.1, PSY1R, PWD | 1 | 1 | MPK11 |
|  | **Hormone pathways** | 17 | 11 | AT4G18450, LECRKA4.1, FRU, CYP81F2, MYB112, 5PTASE11, OFP15, AT5G10120, AT5G20820, COR413IM1, AT2G37030, PDF1.2b, AT4G39670, GA3OX1, AT3G28580, SEN1, AT1G78410 | 12 | 16 | SAUR26 AT4G18450 AT5G55450 PDF1.2b AT3G07310 AT3G51450 MYB112 5PTASE11 AT1G29450 MPK11 MYB7 FKD1 |
|  | **Cellular protein modification** | 12 | 8 | AT1G61480 AT4G17660 AT5G02760 AT5G10120 AT5G46080 AT5G57670 B120 CRK4 CRK41 CRK6 PSY1R PUB54 | 3 | 4 | AT5G10700 CRL MPK11 |
|  | **Transcription factors** | 18 | 11 | AT1G10585 AT3G12910 AT3G25790 AT4G18450 AT4G20970 AT5G10120 AT5G44260 FRU GATA4 HB21 HEC1 HEC2 MYB112 MYR2 NGA4 PI SEP3 SNZ | 6 | 8 | AT1G10585 AT4G18450 AT4G20970 DREB2A MYB112 MYB7 |
| Metabolism | **Nucleo-base containing compound metabolic process** | 25 | 16 | AT1G10585 AT1G17960 AT1G18130 AT3G12910 AT3G25790 AT4G18450 AT4G20970 AT5G10120 AT5G44260 DMT2 FCA FRU GATA4 HB21 HEC1 HEC2 HEI10 KNATM MYB112 NGA4 OFP15 OFP16 PI SEP3 SNZ | 11 | 14 | AT1G10585 AT2G27290 AT4G18450 AT4G20970 AT5G08460 AT5G64420 DREB2A MPK11 MYB112 MYB7 UMAMIT23 |
|  | **Protein metabolic process** | 17 | 11 | AT1G17960 AT1G18130 AT1G61480 AT4G17660 AT4G18450 AT5G02760 AT5G10120 AT5G46080 AT5G57670 B120 CRK4 CRK41 CRK6 FAB1C MPC PSY1R PUB54 | 6 | 8 | AT4G18450 AT5G10700 CRL DREB2A MPC MPK11 |
|  | **Lipid metabolic process** | 9 | 6 | AT3G02620 AT3G52070 AT5G10120 AT5G24200 FAB1C FTM1 GA3OX1 ORP4C RXW8 | 5 | 6 | 5PTASE11 AT2G27290 AT4G16070 AT5G08460 AT5G24200 |
|  | **Secondary metabolic process** | 8 | 5 | AT1G76470 AT5G14700 BCAT-2 CYP81F2 ELIP2 FAR1 GRP3S UGT72E1 | 3 | 4 | GPX7 PPT2 RALFL22 |
|  | **Carbohydrate metabolic process** | 7 | 4 | AT4G19750 AT5G66150 DIN2 FRA8 GolS6 PCK2 QQS | 3 | 4 | BAM6 DIN2 GolS6 |
|  | **DNA metabolic process** | 2 | 1 | DMT2 HEI10 | 1 | 1 | AT5G64420 |
| Stress related | **Abiotic stress** | 18 | 11 | AT4G18450 AT4G39670 AT5G10120 AtLEA4-1 COR413IM1 CYP81F2 CYP82C2 DIN10 DR4 ELIP2 FAR1 FTM1 GA3OX1 GATA4 LECRKA4.1 MGL MYB112 TSA1 | 11 | 14 | AT1G16850 AT4G17250 AT4G18450 AT4G28030 CEST DREB2A GPX7 MPK11 MYB112 MYB7 TAF2 |
|  | **Biotic stress** | 14 | 9 | ACD6 AIG1 AT1G78410 AT3G04210 AT4G20970 AT4G39670 AT5G57670 B120 CRK4 CYP81F2 FTM1 LECRKA4.1 NHL3 TSA1 | 6 | 8 | ADF4 AT3G51450 AT4G20970 AT5G55450 MPK11 TI1 |
| Transport | **Transport facilitation** | 19 | 12 | ABCC3 AIG1 AT1G10585 AT1G78410 AT2G16990 AT3G48970 AT4G22520 AT4G39670 AT5G26690 B120 COPT2 EXO70H4 FRU INT2 JRG21 LECRKA4.1 PUP1 SWEET12 UGT72E1 | 8 | 10 | AT1G10585 AT4G28030 AT5G55450 AT5G55460 JRG21 MPK11 PPT2 SULTR3;1 |
| Reproduction | **Flower development** | 8 | 5 | AT1G17960 AT4G36600 FCA HEC1 HEC2 NGA4 PI SEP3 | 0 | 0 |  |

%= Represent percentage of corresponding functional group (responsive genes involved in specific biological function/all expressed genes of that category)*100. GO enrichment was performed in AgriGO (FDR correction and Fisher’s exact test<0.05) using the whole *Arabidopsis* genome as the background/reference.
