# Supplementary material for: Analysis of peptide PSY1 responding transcripts in the two Arabidopsis plant lines: wild type and psy1r receptor mutant
Source: BMC Genomics. 2014 Jun 6;15(1):441. doi: 10.1186/1471-2164-15-441 (PMC4070568; doi:10.1186/1471-2164-15-441)
Supplement: Supplementary file 5 — Additional file 5: Table S5: Primers used for RT-PCR validation of selected genes identified by microarray after PSY1 treatment in wild type plants. (DOCX 19 KB) [file 12864_2013_6150_MOESM5_ESM.docx]

**Supplementary table 5:** Primers used for RT-PCR validation of selected genes identified by microarray after PSY1 treatment in wild type plants

| **Gene Name** | **Gene locus** | **Specific Primer** |
| --- | --- | --- |
| WVD2 | [At5g28646](http://atted.jp/data/locus/At5g28646.shtml) | ***F: 5’- ATTGTTCTGTTGCTTCTTC-3’***  ***R: 5’- TGCTTCTTGTTCTTCCTTT-3’*** |
| 5PTASE11 | At1g47510 | ***F: 5’- CATCTTCAATCTGTTCTT-3’***  ***R: 5’- ATCTTATGGCTTGTATCA-3’*** |
| RALF35 | At2g32785 | ***F: 5’- GTGTCGCTCTACTGATTA-3’***  ***R: 5’- TGGCTTCGTATATGGATT-3’*** |
| AML2 | [At2g42890](http://atted.jp/data/locus/At2g42890.shtml) | ***F: 5’- AATAGAAGCGAGATAGGT-3’***  ***R: 5’- GTGGAGAATTAGCAACAT-3’*** |
| CRL | At5g51020 | ***F: 5’- TTACTTCAACATAAGAATGC-3’***  ***R:5’- AACTTACCTCAACATCAC-3’*** |
| Hypothetical protein | At1g10585 | ***F: 5’- TGAATGATAGGACCACTTAC-3’***  ***R: 5’- GCGTTAGCGTAGACTTAT-3’*** |
| SCRL3 | [At1g08695](http://atted.jp/data/locus/At1g08695.shtml) | ***F: 5’- AAGGTGCGAACAAGAGGTGT-3’***  ***R: 5’- ACACGTTGGGGAGGTAATGA-3’*** |
| Small auxin responsive protein | At1g29450 | ***F: 5’- CGCTAAGTTACCTGAGTA-3’***  ***R: 5’- GCACTAGAGATTGACATTA-3’*** |
| Small auxin responsive protein | At3g03850 | ***F: 5’- AGAGATGGAGGAGTATTC-3’***  ***R: 5’- CTAAGTCGTCAAGTGATAT-3’*** |
| ATMPK11 | At1g01560 | ***F: 5’- CAAGAAGATTGGTAATGC-3’***  ***R: 5’- GGATAATGTGGTGAAGAT-3’*** |
| RALF33 | At4g15800 | ***F: 5’- ACCGTCCACTTCTTATTC-3’***  ***R: 5’- GTTGATCTCAGAGTCCAT-3’*** |
| RALF22 | At3g05490 | ***F: 5’- TGGCGATAGTAATCTCAG-3’***  ***R: 5’- AACTCCATCTCTTCTTCTT-3’*** |
| Actin2 | At3g18780 | ***F: 5’- TGAGAGATTCAGATGCCCAGAA-3’***  ***R: 5’- TGGATTCCAGCAGCTTCCAT-3’*** |
