# Supplementary material for: Analysis of peptide PSY1 responding transcripts in the two Arabidopsis plant lines: wild type and psy1r receptor mutant
Source: BMC Genomics. 2014 Jun 6;15(1):441. doi: 10.1186/1471-2164-15-441 (PMC4070568; doi:10.1186/1471-2164-15-441)
Supplement: Supplementary file 6 — Additional file 6: Figure S1: The ACT2 (At3g18780) gene expression. The expression of ACT2 was not significantly altered in both plant lines treated with the PSY1 peptide compared to the untreated plant lines. The significance level was tested among three independent biological replicates (n = 3). The scale bars represent standard error (S.E) among three biological samples. (PDF 383 KB) [file 12864_2013_6150_MOESM6_ESM.pdf]

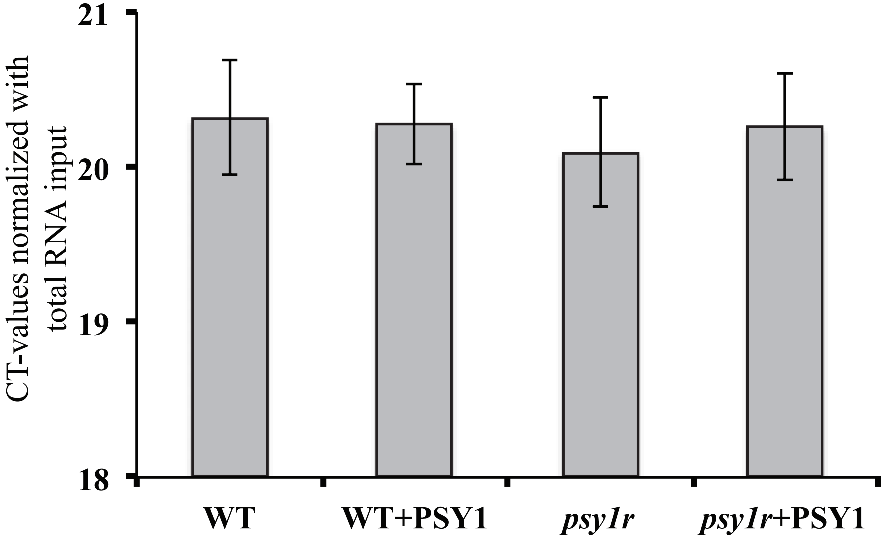

Supplementary Fig 1: **The ACT2 (At3g18780) gene expression.** The expression of ACT2 was not significantly altered in both plant lines treated with the PSY1 peptide compared to the untreated plant lines. The significance level was tested among three independent biological replicates (n=3). The scale bars represent standard error (S.E) among three biological samples.
